# Proangiogenic effect and underlying mechanism of holmium oxide nanoparticles: a new biomaterial for tissue engineering

**DOI:** 10.1186/s12951-024-02642-x

**Published:** 2024-06-21

**Authors:** Yuxiao Luo, Yifan Zheng, Ziwei Chen, Minhua Mo, Jiling Xie, Xiaohe Zhou, Yupeng Wu, Qiyuan Yang, Manjia Zheng, Xiaowen Hu, Liangjiao Chen, Zedong Lan

**Affiliations:** 1 Shenzhen Stomatological Hospital, Southern Medical University, Shenzhen, 518001 Guangdong People’s Republic of China; 2 Department of Orthodontics, School and Hospital of Stomatology, Guangdong Engineering Research Center of Oral Restoration and Reconstruction & Guangzhou Key Laboratory of Basic and Applied Research of Oral Regenerative Medicine, Guangzhou Medical University, Guangzhou, China

**Keywords:** Holmium oxide nanoparticles, Angiogenesis, Migration, EphrinB2, CDC42

## Abstract

**Background:**

Early angiogenesis provides nutrient supply for bone tissue repair, and insufficient angiogenesis will lead tissue engineering failure. Lanthanide metal nanoparticles (LM NPs) are the preferred materials for tissue engineering and can effectively promote angiogenesis. Holmium oxide nanoparticles (HNPs) are LM NPs with the function of bone tissue “tracking” labelling. Preliminary studies have shown that HNPs has potential of promote angiogenesis, but the specific role and mechanism remain unclear. This limits the biological application of HNPs.

**Results:**

In this study, we confirmed that HNPs promoted early vessel formation, especially that of H-type vessels in vivo, thereby accelerating bone tissue repair. Moreover, HNPs promoted angiogenesis by increasing cell migration, which was mediated by filopodia extension in vitro. At the molecular level, HNPs interact with the membrane protein EphrinB2 in human umbilical vein endothelial cells (HUVECs), and phosphorylated EphrinB2 can bind and activate VAV2, which is an activator of the filopodia regulatory protein CDC42. When these three molecules were inhibited separately, angiogenesis was reduced.

**Conclusion:**

Overall, our study confirmed that HNPs increased cell migration to promote angiogenesis for the first time, which is beneficial for bone repair. The EphrinB2/VAV2/CDC42 signalling pathway regulates cell migration, which is an important target of angiogenesis. Thus, HNPs are a new candidate biomaterial for tissue engineering, providing new insights into their biological application.

**Supplementary Information:**

The online version contains supplementary material available at 10.1186/s12951-024-02642-x.

## Article highlights

Holmium oxide nanoparticles (HNPs) promote angiogenesis in vitro and in vivo.

HNPs increase cell migration by mediate filopodia extension, thus promote angiogenesis.

HNPs interact with EphrinB2 to promote its phosphorylation.

EphrinB2 interacts with and activates VAV2.

VAV2 activates CDC42 to promote endothelial cell migration.

**Supplementary Information:**

The online version contains supplementary material available at 10.1186/s12951-024-02642-x.

## Graphical abstract

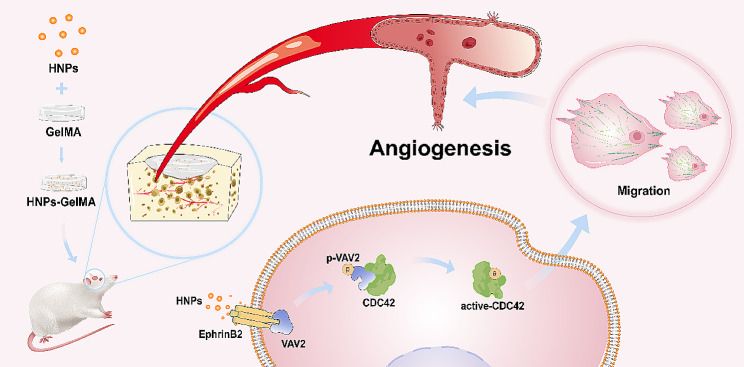

## Background

The reconstruction of large bone defects, secondary to severe trauma, tumour resection, inflammation, and congenital deformities, has been a major challenge [1]. Early comprehensive vascular reconstruction is an effective strategy for increasing the efficacy of bone regeneration and a prerequisite for the long-term functional survival of newly formed bone after biomaterial implantation [2]. Consequently, tissue engineering biomaterials that optimize angiogenesis are actively being developed.

In recent years, researchers have focused on loading various stem cells or cytokines on biological scaffolds to promote angiogenesis [3, 4]. However, biologically active components loaded onto composite materials face challenges such as instability, degradation, and difficult delivery [5]. Nanoparticles themselves may modulate cellular biological processes. Therefore, the development of multifunctional nanoparticles with angiogenic properties to replace these unstable bioactive factors will facilitate the development of regenerative medicine.

Lanthanide metal nanoparticles (LM NPs) possess superior stability and limited immunogenicity, and some of these materials have recently shown good angiogenic effects [6]. Cerium oxide nanoparticles [7], lanthanum oxide nanoparticles [8], and terbium hydroxide nanorods [9] have been shown to promote angiogenesis by regulating intracellular reactive oxygen species (ROS). Holmium oxide nanoparticles (HNPs), another type of LM NPs, have antibacterial activity and can be used as biomarkers [10, 11]. Previous research has shown that alloys or bioactive glasses doped with holmium has increased osteogenic capabilities and biocompatibility [12, 13]. Therefore, HNPs may share the ability to promote angiogenic and osteogenic coupling with other LM NPs, for example, through the formation of H-type vessels. Research on the proangiogenic effects of LM NPs has predominantly focused on the determination of redox capabilities and the observation of neovascularization. However, studies investigating the localization of nanoparticles within endothelial cells and their regulatory effects on endothelial cell functions are lacking.

Angiogenesis is an intricate and dynamic process that includes the directional sprouting of endothelial tip cells, the migration of endothelial cells, lumen maturation, and the establishment of new branches [14]. Endothelial cell migration is a key process that depends on the dynamic reorganization of the cytoskeleton [15]. For example, graphene oxide (GO) nanoparticles can change cell polarity through cytoskeletal reorganization, forming a front end in the direction of migration and a posterior end in the direction of backwards retraction [16]. After nanoparticle implantation, the cell membrane is the first barrier for nanoparticles in the interaction with endothelial cells. The interaction between nanoparticles and the cell membrane inevitably influences the structure and function of the cell membrane, inducing downstream cellular responses. The activation of membrane proteins, such as integrin receptors [17] and VEGF receptors [18], can promote cytoskeletal reorganization. LM NPs (such as cerium oxide nanoparticles) have been reported to activate bone morphogenetic protein receptors(BMPRs) and calcium ion channels in the cell membrane [19, 20]. Additionally, whether the biological activity of HNPs is caused by interactions with membrane proteins is unclear.

In this study, we investigated whether HNPs are novel biomaterials with angiogenic properties. HNPs effectively promoted early angiogenesis in the rat cranial defect area, and the concomitant superior bone regeneration may be attributed to H-type vessels. Additionally, HNPs stimulate endothelial cells to extend filopodia, increase cell migration, and promote tube formation. Further experiments were performed to investigate the cellular mechanisms that govern angiogenesis. HNPs activate the EphrinB2/VAV2/CDC42 cascade, ultimately regulating the migration of endothelial cells and effectively promoting angiogenesis. In summary, this research demonstrates the angiogenic properties and underlying mechanism of HNPs, providing a basis for their application in tissue engineering repair.

## Materials and methods

### Characterization of HNPs

HNPs were purchased from Zhongkekeyou Nano Technology (Beijing, China). The size and morphology were determined by scanning electron microscopy (SEM, TESCAN MIRA LMS, Czech Republic) and transmission electron microscopy (TEM, FEI Tecnai G2 F20, USA). The elemental composition was identified through energy-dispersive X-ray spectroscopy (EDS) analysis. The zeta potential was evaluated by a Zetasizer Nano-ZS system (Malvern Zetasizer Nano ZS90, UK). The structural characteristics of the HNPs were revealed through X-ray diffraction (XRD) using SmartLab SE (Rigaku SmartLab SE, Japan). The functional and vibrational characteristics of the HNPs were identified through Fourier transform infrared spectroscopy (FTIR, Nicolet iN10, USA) and Raman spectroscopy (LabRam HR Evolution, France).

### In vivo studies

#### Rat cranial defect model

Gelatine methacryloyl (GelMA, EFL-GM-90, Suzhou Intelligent Manufacturing Research Institute, China) was prepared at 10% (w/v) and then mixed with HNPs at different ratios (0.0001 wt%, 0.0005 wt%, 0.001 wt%, and 0.005 wt%). Each mixture was transferred to a circular mould measuring 1 mm in depth and 5 mm in diameter, then exposed to visible light at 405 nm for 1 min for curing.

Guangdong Huawei Micro Detection Co., LTD. (China) provided 6-week-old male Sprague-Dawley rats for in vivo studies. 48 rats were randomly divided into four groups, each containing 6 rats (*n* = 6): the control group (not implanted), the GelMA group (implanted with GelMA), the HNPs-GelMA group (implanted with 0.0001/0.0005/0.001/0.005 wt% HNPs-GelMA) and the 0.001 wt% HNPs-GelMA + ML141 (10 µM, Cat# S7686, Selleck, USA) group. Under pentobarbital sodium anaesthesia, circular full-layer bone defects of critical size were created on both sides of the skull using a ring bone drill with an internal diameter of 5 mm. The implants were sutured. The subsequent experiments were conducted two weeks later. The Animal Ethics Committee of Guangdong Huawei Micro Detection Co., Ltd. approved all procedures conducted in this study (202304011).

#### Three-dimensional (3D) images and microcomputed tomography (micro-CT) analysis

After the rats were anaesthetized, rhodamine B (Cat# R8881, Sigma, Germany) was injected intravenously through the inner canthus. A multiphoton laser scanning microscope (FV1200MPE, Olympus, Japan) was used to observe vascular formation in the cranial defect area of the rats, and assessment was conducted using Imaris 9.0.1. Subsequently, the rats were euthanized by being placed in sealed cages and continuously filling the cages with carbon dioxide.

For micro-CT analysis, the cranial defect area was dissected along with the hydrogel. After 4% paraformaldehyde was added for 3 days, the 3D morphology was assessed by micro-CT (BrukerSkyScan1275x, Germany) at a current of 78 µA and a voltage of 48 kV. The scanning accuracy was 10 microns. Images of the bone defect area were reconstructed based on the micro-CT findings using MIMICS software.

#### Histological analysis

The cranial defect area samples were collected, fixed with 4% paraformaldehyde (Cat# BL539A, Biosharp, China) for 24 h, decalcified in 10% EDTA (Cat# DD0002, Leagene, China) for 1 month, and embedded in paraffin wax. The sample was sliced into continuous sections with a thickness of 4 μm for subsequent histological evaluation.

Following the manufacturer’s instructions, consecutive cross-sectional slices of the decalcified samples were subjected to haematoxylin and eosin (HE) staining and immunohistochemistry for endothelial cell adhesion molecule 1 (CD31, 1:20, Cat# AF3628, R&D, USA). Additionally, immunofluorescence costaining experiments were performed on tissue sections using primary antibodies against CD31 (1:20, Cat# AF3628, R&D, USA) and Emcn (1:100, Cat# sc-65495, Santa Cruz, USA), as well as secondary antibodies Cy3-conjugated donkey anti-rabbit IgG (1:200, Cat# GB21403, Servicebio, China) and FITC-conjugated donkey anti-goat IgG (1:200, Cat# GB22404, Servicebio, China). Images of the immunofluorescence costained samples were taken via confocal microscopy. The proportion of H-type vessels’ area was then assessed using ImageJ in randomly selected areas.

### In vitro experiments

#### Cell culture

Human umbilical vein endothelial cells (HUVECs) were obtained from Cyagen Biosciences, Inc. (Cat. #HUVEC-20,001, Cyagen, China), cultured in endothelial cell medium (ECM, Cat# 1001, ScienCell, USA) supplemented with 5% foetal bovine serum (FBS, Cat. #0025, ScienCell, USA), 1% penicillin/streptomycin (P/S, Cat. #0503, ScienCell, USA) and 1% endothelial cell growth supplement (ECGS, Cat. #1052, ScienCell, USA), and then incubated in incubators (5% CO_2_, 37 °C). HUVECs from the third to sixth generations were used in the subsequent experiments.

#### Cell proliferation

HUVECs were inoculated in 96-well plates and incubated with HNPs at concentrations of 0, 1, 10, 100 or 1000 ng/mL for 12, 24 or48 h. 10 µL of Cell Counting Kit-8 (CCK-8, Cat# CK04, Dojindo Laboratories, Japan) solution was added to each well, and the cultures were incubated at 37 °C for 2.5 h. After centrifugation, the supernatant was gathered and the absorbance at 450 nm was assessed using VersaMax Molecular Devices (USA).

#### Cell migration

For the scratch assay, HUVECs were inoculated in a 6-well plate. After the HUVECs had grown to 100% confluence, a sterile 200 µL pipette tip was used to make cell-free scratches in the middle of each well. PBS was used to rinse off the nonadherent cells, and 0 or 10 ng/mL HNPs prepared from serum-free ECM were added to each well. Images were taken at 0, 12 and 24 h, and the remaining gap areas were quantitatively analysed using ImageJ.

In the Transwell experiment, the treated HUVECs were inoculated into the upper cavity of Transwell cell culture inserts (Cat# 3422, Corning, China) at 2 × 10^4^ cells per well and cultured with 200 µL of ECM (serum free). Then, 600 µL of ECM was added to the lower cavity. 24 h later, the cells that migrated to the lower cavity were fixed with 4% paraformaldehyde for 20 min and then subjected to crystal violet staining (Cat# DZ0055, Leagene, China) for 25 min. The images of each group were obtained by inverted microscope and the counts of cells migrating into the lower chamber were quantified by ImageJ software.

#### Tube formation

HUVECs were inoculated in 12-well plates and treated with 0 or 10 ng/mL HNPs. Matrigel (Cat# 356,231, BD Bioscience, USA) was added to the ibidi slides (Cat# 81,506, Ibidi, Germany), followed by a 30-minute incubation at room temperature. Then, the slides were transferred to a 37 °C incubator for an additional 30 min. Subsequently, 50 µL of 5 × 10^3^ HUVECs was added to both groups of media and seeded into the ibidi plates coated with Matrigel. After 4 h, the cells were photographed with a stereomicroscope (Leica, Germany), and the numbers of nodes, meshes and the master segment length were quantitatively analysed with ImageJ.

#### Immunofluorescence staining

HUVECs were fixed with 4% paraformaldehyde (Cat# DF0135, Leagene, China) for 20 min, and the membranes were disrupted with 0.5% Triton X-100 (Cat# T6200G, Biotopped, China) for 5 min and then blocked with 5% immunostaining blocking solution (Cat# P0260, Beyotime, China) for 1 h. After washes with PBS, the HUVECs were incubated overnight with primary antibodies against EphrinB2 (1:100, Cat# AF6343, Affinity, USA) and VAV2 (1:100, Cat# 21924-1-AP, Proteintech, USA) at 4 °C. Subsequently, the HUVECs were incubated with FITC-labelled goat anti-rabbit IgG (1:200, Cat# GB22303, Servicebio, China) and Cy5-labelled goat anti-mouse IgG (1:200, Cat# GB27301, Servicebio, China) secondary antibodies at room temperature for 1 h. The cells were then incubated with FITC-labelled phalloidin (1:150, Cat# CA1620-300T, Solarbio, China) at 37 °C for 30 min. Finally, the cells were incubated with DAPI staining solution containing antifade mounting medium (Cat# G1407-25ML, Servicebio, China) for 20 min and imaged using laser confocal microscopy (Stellaris 5, Leica, Germany).

For preparation of FITC-HNPs, BSA-FITC solution (Cat# SF063, Solarbio, China) was mixed with HNPs at a ratio of 1:10, followed by incubation for 8 h in a dark incubator at 37 °C. After the mixture was centrifuged at 14,000×g at 4 °C for 35 min, the collected FITC-HNPs samples were resuspended in the ECM.

#### RNA-seq assay

Total RNA was extracted from HUVECs exposed to 0 or 10 ng/mL HNPs using TRIzol reagent (Cat# 15596026, Thermo Fisher, USA). The purity of the extracted samples was evaluated using a NanoDrop spectrophotometer (IMPLEN, CA, USA). Once the samples were deemed suitable for detection, 1 to 3 µg of total RNA was extracted from each sample to construct the transcriptome sequencing library. Transcriptome sequencing and data analysis were conducted on an Illumina NovaSeq 6000 S4 platform.

#### Reverse transcription‒polymerase chain reaction (RT-PCR)

Total RNA was extracted using the SteadyPure Universal RNA Extraction Kit (Cat# AG21017, Accurate Biology, China). Followed by reverse transcription using the Evo M-MLV Reverse Transcription Kit (Cat# AG11706, Accurate Biology, China). RT-qPCR was performed using the SYBR Green Pro Taq HS Premix qPCR Kit (Cat# AG11701, Accurate Biology, China). The primer sequences utilized in this experiment are depicted in Table S1. The relative expression of VEGF, CD31 and EFNB2 mRNA were normalized to the expression level of glyceraldehyde-3-phosphate dehydrogenase (GAPDH).

### Western blotting (WB)

Cells were seeded at a density of 6 × 10^5^ cells per well in a 6-well plate and treated with 10 ng/mL HNPs or 10 µmol/mL holmium solution (Cat#H117331, Aladdin, China). Cell proteins were extracted from cells that reached 90% confluence using RIPA lysis buffer (Cat# P0013B, Beyotime, China) with PMSF (Cat# ST506, Beyotime, China) and phosphatase inhibitor (Cat#CW2383S, CWBIO, China). The protein concentration extracted from the samples was determined using the BCA Protein Assay kit (Cat# P0010, Beyotime, China). Then, protein samples from each group were separated by SDS‒polyacrylamide gel electrophoresis (Cat# P0014D, Beyotime, China) and transferred onto a PVDF membrane, which was blocked with 5% skim milk powder (Cat# P0216, Beyotime, China) at 25℃ for 1 h. Antibodies against CD31 (1:1000, Cat# 11265-1-AP, Proteintech, USA), VEGF (1:1000, Cat# 19003-1-AP, Proteintech, USA), EphrinB2 (1:1000, Cat# sc-398,735, Santa, USA), phosphorylated EphrinB2 (Tyr304) (1:1000, Cat# AF7321, Affinity, China), VAV2 (1:1000, Cat# 21924-1-AP, Proteintech, USA), phosphorylated VAV2 (Tyr142) (1:1000, Cat# AF4446, Affinity, China), CDC42 (1:1000, Cat# 10155-1-AP, Proteintech, USA), and GAPDH (1:1000, Cat# 10494-1-AP, Proteintech, USA) were then incubated with the membranes overnight at 4 °C. Following TBST washes, the membrane was incubated with HRP-conjugated anti-rabbit IgG (1:5000, Cat# 7074P2, CST, USA) or anti-mouse IgG (1:5000, Cat# 7076P2, CST, USA) at 25℃ for 1 h. The proteins on PVDF membranes were visualized using a high-sensitivity chemiluminescence kit (Cat# P0018AS, Beyotime, China).

### Immunoprecipitation (IP) and co-immunoprecipitation (CO-IP)

IP experiments were used to determine the expression of active CDC42. HUVECs were cultured in 10 cm culture dishes. Upon reaching 90% confluence, the cells were treated with corresponding reagents according to different groups. After 24 h, the cells were lysed with lysis buffer at 4 °C for 30 min, followed by centrifugation at 4 °C for 30 min to obtain the supernatant. Subsequently, active CDC42 antibody (Cat# 26905, WuHan NewEast Biosciences Co., Ltd., China) and protein G beads were added and incubated at 4 °C for 1 h. Afterwards, the beads were washed three times with lysis buffer. After the last wash, all the supernatant was carefully removed. The samples were resuspended in 20 µL of 2x loading buffer and boiled for 5 min at 100 °C, the supernatant was aspirated and retained. The final samples were analysed by protein blotting using an anti-CDC42 antibody.

CO-IP assays conducted with a magnetic immunoprecipitation kit (Cat# Bes3011, Biotech Group Co., Ltd., China), and the results were visualized via WB. Protein samples from HUVECs transfected with the EFNB2 and VAV2 overexpression plasmids were extracted in 10 cm culture dishes, and then, 500 µL of the supernatant was incubated with 8 µg of IP antibodies (EphrinB2 or VAV2) or control IgG at 4 °C for 12 h. In the mixture obtained from the previous step, add magnetic beads and incubate at 4 °C for 2 h. Wash the magnetic beads four times with lysis buffer and discard the supernatant. Add elution buffer and boil the mixture for 10 min. Retain the supernatant, then add 5x loading buffer and boil for 5 min. Finally, WB experiments were carried out.

### Transfection

HUVECs were placed in 10 cm culture dishes at a density of 10 × 10^5^ cells/well. Lipofectamine 3000 (Cat# L3000001, Thermo Fisher, USA) in Opti-MEM I reduced serum medium (Cat# 31,985,070, Thermo Fisher, USA) was used to transiently transfect siEFNB2, siVAV2 and the overexpression plasmids pcDNA3.1(+)-EFNB2-3xFLAG and pcDNA3.1(+)-VAV2-HA. The siRNAs used in this study were designed and constructed by OBiO Technology Co., Ltd. (China) (Table S2).

### Analysis of ROS

HUVECs were inoculated in 6-well plates at a density of 5 × 10^5^ cells/well and cultured with ECM containing 0, 1, 10, 100 or 1000 ng/mL HNPs for 24 h. Each sample was incubated with 10 µM DCFH-DA (Cat# S0033S, Beyotime, China) for 20 min, followed by three washes with PBS. The samples were resuspended in PBS and immediately analyzed for ROS levels using a flow cytometer (BD FACSAria III, USA).

In addition, the positive control group was treated with active oxygen positive control reagent for 30 min. One millilitre of serum-free ECM containing 10 µM DCFH-DA was added to each well and incubated in the cell incubator in the dark for 20 min. After washing the cells three times with PBS, 1 mL of ECM (serum free) was added to each well, followed by immediate image capture using a laser scanning confocal microscope.

### Molecular dynamic (MD) simulations

This study obtained the structure files for EphrinB2 and VAV2 from the PDB database. Protonation under neutral conditions was initially carried out using an H + + 3 online server. Pymol software was then used to remove the heteroatoms and water molecules from the crystal structure, leaving only the protein structure. HawkDock was used for interconnection, and the configuration with the highest initial interconnection score was selected for further analysis. The configuration with the highest interconnection score was selected from among the interconnection configurations for calculation by the MMGBSA of Hawdock. PyMOL 2.04 and ChimeraX were used for 3D graphic analysis, and ligplus + was used for two-dimensional effect analysis [21, 22].

### Statistical analyses

All the data are expressed as the mean ± standard deviation (SD) with a minimum sample size of ≥ 3. Statistical comparisons were performed using univariate analysis of variance (ANOVA) or t tests (if applicable) with GraphPad Prism software. *P* < 0.05 was considered to indicate statistical significance.

## Results

### Characterization of HNPs

**Fig. 1:**
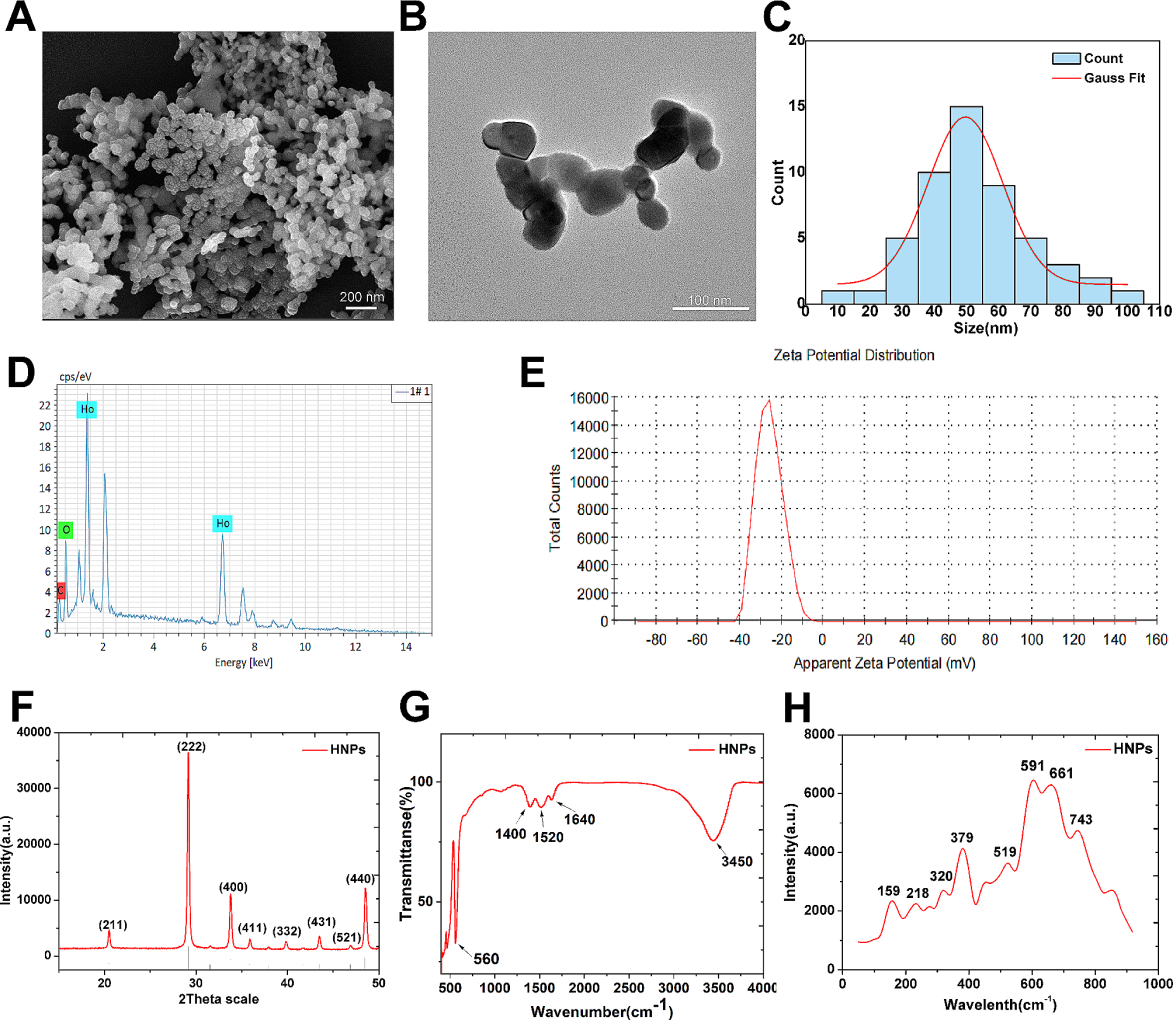
Characterization of HNPs. (**A**) SEM image of HNPs. (**B**) TEM image of HNPs. (**C**) Particle size distribution diagram, and the average particle size of the HNPs is approximately 50 nm. (**D**) EDS elemental analysis spectrum. (**E**) Zeta potential of HNPs in neutral PBS solution. (**F**) XRD of HNPs. (**G**) FTIR of HNPs. (**H**) Raman spectrum of HNPs

This study employed various methods to characterize the properties of HNPs. As we aimed to elucidate the biological mechanisms triggered by the physicochemical properties of HNPs themselves, no additional modifications were made to the HNPs. SEM and TEM showed that the HNPs exhibited uniform spherical or cubical shapes (Fig. 1A, B). The average particle size, as determined from the TEM results, was approximately 50 nm (Fig. 1C), indicating that the particles were nanomaterials. The EDS results confirmed the presence of holmium and oxygen (Fig. 1D). The zeta potential was approximately − 27.9 mV (Fig. 1E), indicating good stability of the HNPs in neutral aqueous solution [23]. The XRD results for the HNPs were consistent with those of previous studies, confirming a standard crystal structure of body-centred cubic Ho_2_O_3_ (Fig. 1F) [24]. The FTIR spectrum showed a strong vibrational peak at 560 cm⁻¹, attributed to traditional metal-oxygen bonds (Ho-O) (Fig. 1G) [25]; vibrations at 1400 cm⁻¹ and 3450 cm⁻¹ were attributed to hydroxyl groups (-OH) and the stretching of hydroxyl groups (-OH). Rare earth oxide materials have complex Raman spectra that vary with the excitation wavelength. Typically, strong Raman peaks of these materials are observed in the range of 320–420 cm⁻¹ [26]. The Raman spectrum obtained at a wavelength of 785 nm revealed a strong peak at 379 cm⁻¹, indicating the presence of well-crystallized HNPs (Fig. 1H) [27]. These results confirmed the crystalline nature and excellent dispersibility of the HNPs used in this study.

### HNPs promoted angiogenesis and optimized osteogenesis in vivo

Angiogenesis is an essential and early phase in the dynamic process of bone regeneration. To explore whether HNPs have a regulatory effect on angiogenesis, we designed a rat cranial bone defect model for observation. Therefore, we utilized GelMA hydrogels as scaffolds loaded with HNPs in vivo and implanted them into a rat cranial bone defect model to investigate whether HNPs can promote early angiogenesis and synergistically facilitate osteogenesis.

**Fig. 2:**
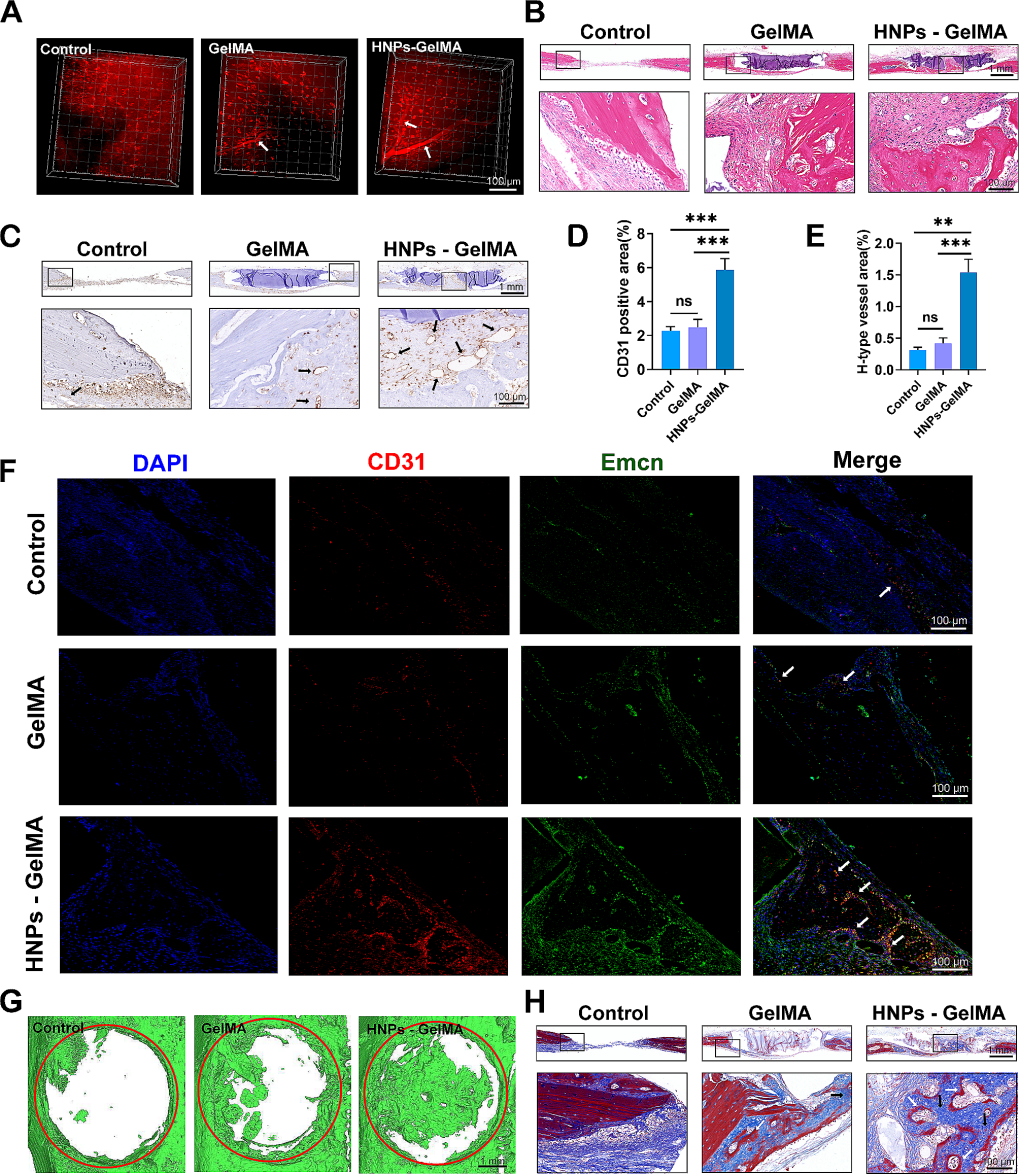
HNPs facilitate angiogenesis in the cranial defect region of rats. (**A**) Three-dimensional images taken by multiphoton confocal microscopy showing vascular generation in the rat cranial bone defect area (the arrows indicate blood vessels). (**B**) HE staining. (**C-D**) CD31 immunohistochemical staining (the arrows indicate blood vessels). Quantitative analysis of positive areas after CD31 immunohistochemical staining. (**E-F**) Immunofluorescence costaining of CD31 (red) and Emcn (green) (the arrows indicate H-type blood vessels). Quantitative analysis of H-type vascular area. (**G**) Micro-CT analysis showing bone formation in the rat cranial bone defect area (the red circle indicates the original defect modelling zone). (**H**) Masson’s trichrome staining (the white arrows indicate newly bone, and the black arrows indicate soft tissue). The data are shown as the mean ± SD; *n* = 6; ns, no significant difference; **P < 0.01; ***P < 0.001

GelMA and HNPs-GelMA with different mass ratios were implanted into a rat cranial bone defect model. Two weeks post-surgery, no animals in any group experienced mortality or significant weight changes (Fig. S1D). In the bone defect area, the multiphoton laser scanning microscopy images showed that the number, volume, and spatial distribution area of newly developed blood vessels in the HNPs-GelMA group were notably greater than those in the control group and the GelMA group (Fig. 2A). The nature and type of these vascular tissues need to be further confirmed by subsequent experiments. Samples from each group were collected from the rat cranial defect area. Tissue sections were used for further observation and analysis. HE staining revealed that the HNPs-GelMA displayed more typical luminal structures, including newly formed single-layer flattened circular structures, increased new bone formation, and a more regular arrangement of connective tissues (Fig. 2B). CD31 is a surface marker for endothelial cells [28]. Immunohistochemical analysis of CD31 further confirmed that these newly formed luminal structures were new blood vessels. The HNPs-GelMA group exhibited a greater number of new vessels, larger luminal volumes, and deeper staining (Fig. 2C, D). H-type vessels are characterized by high levels of CD31 and Emcn, which are important markers of endothelial and osteocyte junctions in bone biology [28]. Vascularization precedes ossification, with the majority of Runx2^+^ cells, Collagen1α^+^ cells, and Osterix^+^ bone progenitor cells selectively near H-type vessels [29]. Consistent with our expectations, staining for CD31 and Emcn revealed a significantly increased density of the H-type vessels in the cranial bone defects of the rats in the HNPs-GelMA group (Fig. 2E, F), suggesting greater and faster new bone regeneration. Furthermore, after micro-CT scanning, we identified a positive correlation between neovascularization and new bone formation. The HNPs-GelMA group exhibited the greatest degree of bone regeneration in the defect area (Fig. 2G). Masson staining revealed that the collagen deposition was significantly increased in the HNPs-GelMA group, and the structure was more regular (Fig. 2H). The Sirius red staining results were similar to those of Masson’s trichrome staining (Fig. S2A), and polarized light images showed that there was more orange-red type I collagen in the HNPs-GelMA group, which was more conducive to new bone formation (Fig. S2B). In conclusion, vascular regeneration in the rat cranial bone defect area was greater in the HNPs-GelMAgroup than in the control group and the GelMA group, which indicated that the addition of HNPs promoted angiogenesis and that bone reconstruction was improved in areas with abundant vessels. The 0.001% HNPs-GelMA group demonstrated the best performance and was used for subsequent in vivo experiments (Figure S1A, B, C).

### HNPs promoted HUVEC tube formation and migration in vitro

**Fig. 3:**
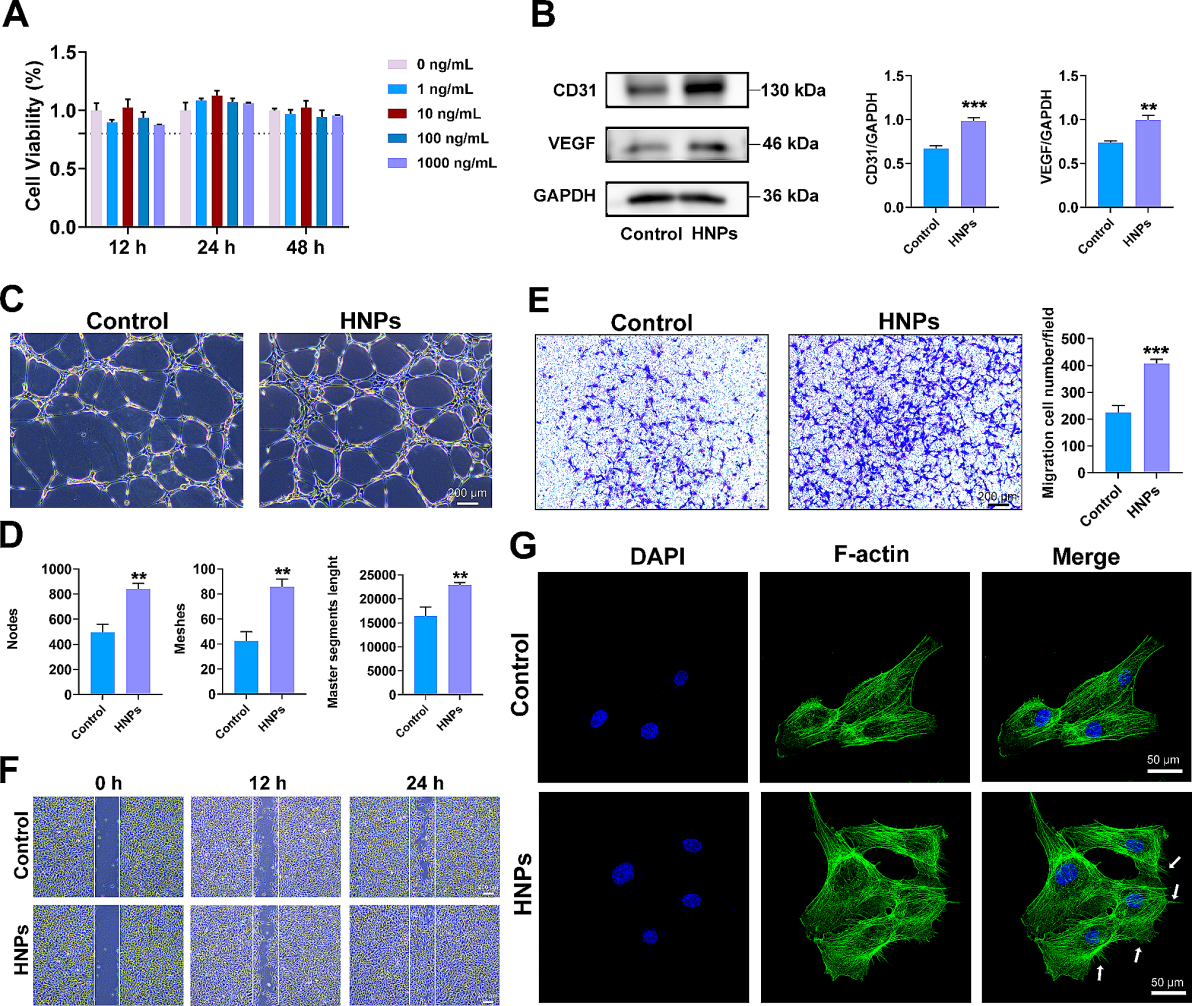
The impact of HNPs on angiogenesis in vitro. (**A**) CCK-8 assay was performed to determine cell viability of HUVECs in different group. (**B**) Protein levels of VEGF and CD31; the results from the quantitative analysis are presented as a histogram. (**C-D**) Tube formation assays of HUVECs. The results of the quantitative analysis of the node count, mesh count, and master segment length are shown in histograms. (**E**) The transwell assay was used to detect the effect of HNPs on HUVECs migration and quantitative analysis was performed. (**F**) The effect of HNPs on HUVECs migration was detected by the scratch test. (**G**) Cytoskeleton staining showed that HUVEC extended more filopodia after HNPs treatment. The data are shown as the mean ± SD; *n* = 3; **P < 0.01; ***P < 0.001

To gain a more comprehensive understanding of the regulatory role of HNPs in angiogenesis, we performed further in vitro studies on HNPs. The biocompatibility of HNPs was first assessed using CCK-8 assays. As shown in Fig. 3A, there was no significant difference in the proliferative capacity of HUVECs. Based on the CCK-8 results, different concentrations of HNPs were used to study the expression of angiogenesis-related genes through RT-qPCR. The RT-qPCR results demonstrated a significant increase in the expression of proangiogenic genes (including VEGF and CD31) at the transcriptional level in the HUVECs cultured with different concentrations of HNPs at 12 and 24 h, and the 10 ng/mL group showing the most significant difference (Fig. S3). Additionally, the protein expression levels of VEGF and CD31 exhibited similar trends (Fig. 3B). Consequently, the 10 ng/mL HNPs group was selected for subsequent in vitro experiments.

Angiogenesis involves the migration of endothelial “tip cells” from existing vessels towards new vessel sprouts, proliferation of stalk cells, formation of lumens, fusion of vessel sprouts, and coverage by perivascular cells to establish perfused new vessels [14]. Therefore, three classic experiments on endothelial cells, including capillary tube formation, migration, and scratch assays, were further conducted. The capillary tube formation assays and quantitative analysis indicated that the HNPs group formed approximately 1.5 times more nodes and meshes than the control group, with significantly longer master segment length than those in the control group, providing direct evidence of increased angiogenesis (Fig. 3C, D). After incubation for 24 h, the number of HUVECs migrating to the lower chamber in the Transwell inserts in the HNPs group was approximately twice that in the control group (Fig. 3E). The scratch experiments revealed that HNPs significantly promoted cell migration (Fig. 3F). However, the detailed molecular regulatory mechanisms require further exploration [30]. The results of these experiments collectively demonstrated that HNPs have a positive effect on angiogenesis, especially in promoting migration.

The continuous reshaping of the actin cytoskeleton of endothelial cells is crucial for their migration during angiogenesis [31]. Therefore, the IF results indicated that the HUVECs cultured with HNPs exhibited more cell protrusions and elongated filamentous pseudopodia than did the control cells (Fig. 3G). Filamentous pseudopodia are long, thin cell protrusions composed of microfilaments, which are typically located at the leading edge of the cell, that guide directional cell movement. Therefore, HNPs promote cell migration by inducing HUVECs to extend more filamentous pseudopodia.

This study provides preliminary evidence that HNPs mediate the extension of filamentous pseudopodia in endothelial cells, promoting cell migration and further regulating vascular regeneration. However, the underlying signalling pathway requires further exploration.

### HNPs induced the extension of filopodia in HUVECs through the activation of VAV2/CDC42

**Fig. 4:**
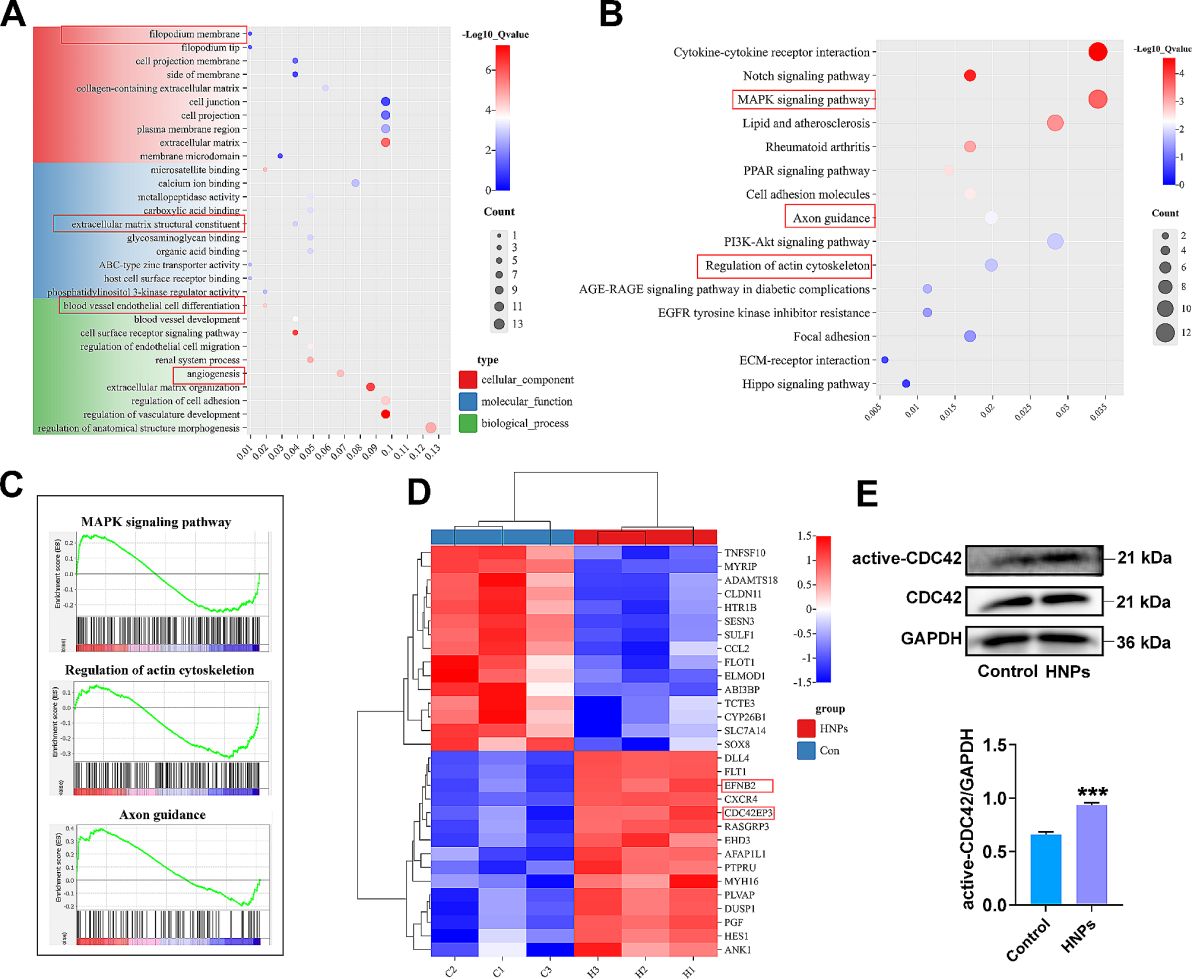
Transcriptomics and WB results confirmed that CDC42 was activated by HNPs. (**A**) GO analysis revealed the enrichment of biological processes, molecular function and cellular component. (**B**) According to KEGG analysis, MAPK signalling pathway, regulation of actin cytoskeleton, and axon guidance were enriched in the differentially expressed genes. (**C**) GSEA analysis confirmed the upregulation of the MAPK signalling pathway, regulation of actin cytoskeleton, and axon guidance by HNPs. (**D**) Heatmap displaying the fold changes in the expression of selected genes. (**E**) The protein expression levels of CDC42 and active-CDC42 in HUVEC treated with 0 or 10 ng/mL for 24 h were measured by WB assay (***P < 0.001)

Previous studies have suggested that certain lanthanide-based inorganic metal nanoparticles, such as lanthanum oxide nanoparticles and neodymium oxide nanoparticles, promote angiogenesis by generating low concentrations of ROS [8, 32]. However, there was no significant difference in the change of ROS within HUVECs after HNPs treatment compared to the control group (Figure S4). This result suggests that HNPs promote angiogenesis through alternative mechanisms. To further explore the potential mechanisms by which HNPs promote angiogenesis, we treated HUVECs with 0 ng/mL or 10 ng/mL HNPs, and RNA was extracted for transcriptome sequencing 24 h later. Transcriptomic analysis provided further insight into the biological effects of HNPs on HUVECs. A total of 104 differentially expressed genes (DEGs) were detected between the HNPs group and the control group, with 54 upregulated and 50 downregulated genes. Gene Ontology (GO) functional enrichment classified DEGs into different categories, which included filopodium membrane, extracellular matrix structural constituent, and angiogenesis (Fig. 4A). Kyoto Encyclopedia of Genes and Genomes (KEGG) and gene set enrichment analysis (GSEA) indicated enrichment in pathways such as the MAPK signalling pathway, regulation of actin cytoskeleton, and axon guidance, suggesting a positive correlation among these pathways (Fig. 4B, C). These results indicate that HNPs modulate HUVECs migration during angiogenesis. Further analysis of genes related to the enriched categories and pathways revealed significant upregulation of CDC42 effector protein 3 (CDC42ep3) expression (Fig. 4D), indicating that the upstream molecule CDC42 is significantly upregulated or activated by HNPs. CDC42 plays a pivotal regulatory role in the cell cytoskeleton, inducing the assembly of actin filaments to form filopodia, thereby promoting endothelial cell migration [33]. The correlation of CDC42 with the results of in vivo and in vitro experiments highlights the impact of HNPs on endothelial cell migration. To confirm whether the expression or status of CDC42 indeed changed in this study, we performed additional Western blot and immunoprecipitation experiments. Consistent with the sequencing results, the active form of CDC42 (active-CDC42) significantly increased in the HNPs group compared to the control group, while the total protein level of CDC42 remained unchanged (Fig. 4E).

**Fig. 5:**
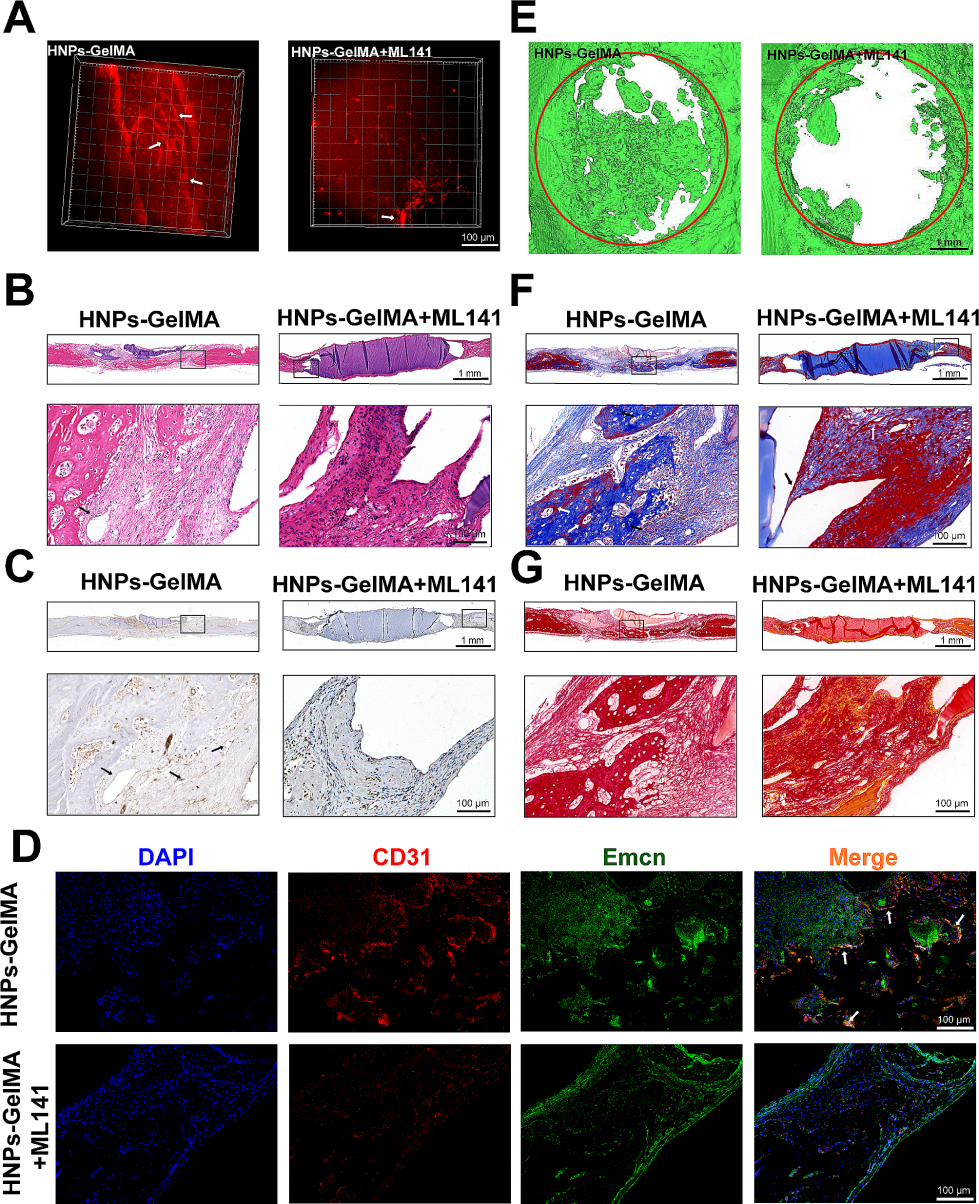
CDC42 plays a role in HNPs-mediated angiogenesis promotion in vivo. (**A**) 3D images of the newly blood vessels taken by multiphoton confocal microscopy (the arrows indicate the newly blood vessels). (**B**) HE staining of the rat cranial bone defect area (the arrows indicate the newly blood vessels). (**C**) CD31 immunohistochemical staining (the arrows indicate the newly blood vessels). (**D**) CD31 (red) and Emcn (green) immunofluorescence costaining. (**E**) Micro-CT analysis (the red circle indicates the original defect modelling zone). (**F**) Masson staining (the white arrows indicate newly bone, and the black arrows indicate soft tissue). (**G**) Sirius red staining showed that collagen deposition was significantly reduced in the rat calvarial defect area after inhibition of CDC42

Subsequently, we investigated the role of CDC42 in mediating angiogenesis in vivo. After addition of the inhibitor ML141, neovascularization decreased, and the vessel structure was more disordered, as shown by the multiphoton confocal microscopy images (Fig. 5A). Quantitative analysis revealed that the area and number of HE and CD31-positive neovascular structures were significantly reduced (Fig. 5B, C; Fig. S5A). Additionally, the newly formed H-type vessels was significantly reduced by approximately 81.2% (Fig. 5D, S5B). This finding suggested that the inhibition of CDC42 significantly impaired the angiogenic ability of HNPs. Furthermore, micro-CT revealed fewer bones in the area of the cranial defect (Fig. 5E). Masson staining and Sirius red staining also demonstrated that collagen deposition was significantly decreased (Fig. 5F, G). Thus, we concluded that HNPs promote angiogenesis through CDC42 and synergistically promote bone regeneration.

**Fig. 6:**
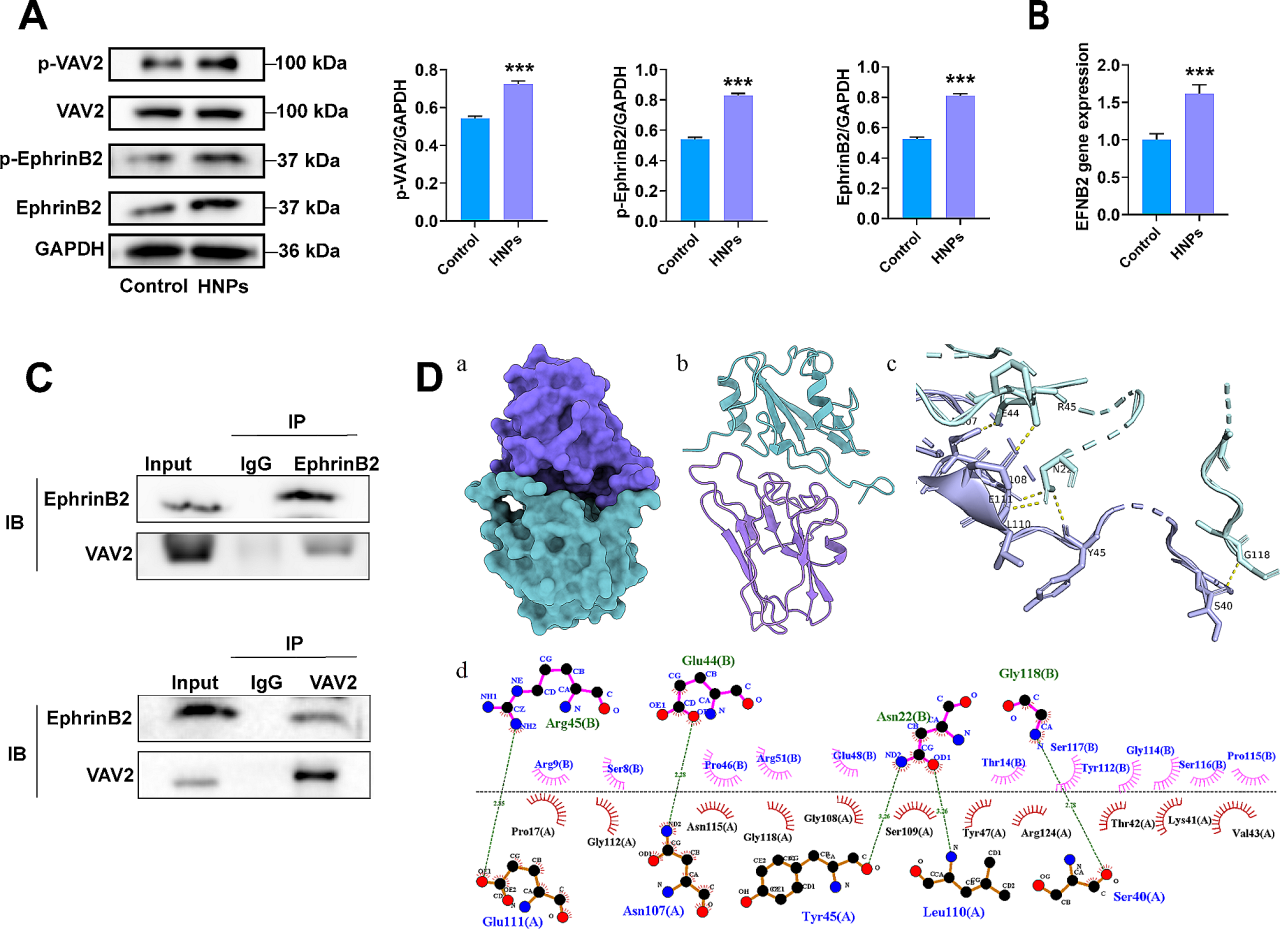
Interaction between EphrinB2 and VAV2. (**A**) WB analysis of the protein expression of p-VAV2, VAV2, p-EphrinB2, and EphrinB2. (**B**) RT‒qPCR determination of EphrinB2 gene expression levels. (**C**) CO-IP experiment was performed to confirm the interaction between EphrinB2 and VAV2. (**D**) MD simulation analysis of the protein-conformation interface interaction between EphrinB2 and VAV2 (a and b are the surface and ribbon structures of the complex; c is the 3d structure of the interface interaction of the complex; d is the 2d interaction interface analysis of the complex), (***P < 0.001)

CDC42 can be activated by guanine nucleotide exchange factors (GEFs), especially VAV2 [34]. After VAV2 knockdown, WB showed that the expression of active CDC42 decreased in the siVAV2 group. This finding indicates that VAV2 can activate CDC42. However, our sequencing results did not reveal significant upregulation of VAV2. Therefore, we validated the expression of VAV2 after HNPs treatment of HUVECs through WB, and the results showed that the total protein level of VAV2 remained unchanged, while the expression of phosphorylated VAV2 significantly increased (Fig. 6A).

In summary, our results indicated that HNPs promote phosphorylation of VAV2 to activate CDC42, exerting biological effects that increase HUVEC migration and, consequently, promote angiogenesis.

### Upregulation and activation of EphrinB2 by HNPs further activate VAV2

VAV2 can interact with various tyrosine-phosphorylated cell surface receptors [35]. Moreover, we observed significant upregulation of the expression of the migration-related membrane protein-encoding gene EFNB2 (gene name for EphrinB2) in the sequencing results (Fig. 4D). EphrinB2 is a transmembrane protein with tyrosine residues in its protein structure. The phosphorylation of tyrosine residues in signalling molecules allows the docking of these signalling molecules, which is relevant for biological processes such as cell migration [36]. Therefore, we hypothesize that EphrinB2 interacts with VAV2 to transduce signals. First, by RT-qPCR and WB analysis, we indicated that the expression of both EphrinB2 and phosphorylated EphrinB2 was significantly upregulated in the HUVECs treated with HNPs (Fig. 6A, B). Then, we performed a CO-IP experiment to confirm the interaction between EphrinB2 and VAV2. The results indicated that when VAV2 was used as the target antigen, EphrinB2 was found on the PVDF membrane, and vice versa (Fig. 6C). This finding suggested that EphrinB2 and VAV2 can interact with each other. For further investigation, we performed MD simulation analysis of the protein-conformational interface interaction between EphrinB2 and VAV2. The combined conformation of EphrinB2 and VAV2 is shown in Fig. 6D. The interface interaction between EphrinB2 and VAV2 involved 16 and 15 amino acid residues, respectively, and the binding hotspot amino acid residues were 10 and 6, respectively, with 5 hydrogen bonds. Among these residues, R-A-Asn-107 and L-B-Arg-45 had the highest binding free energies. The values were − 5.71 and − 4.92 kcal/mol, indicating these residues may play crucial roles in the binding of EphrinB2 to VAV2 (Table S3).

**Fig. 7:**
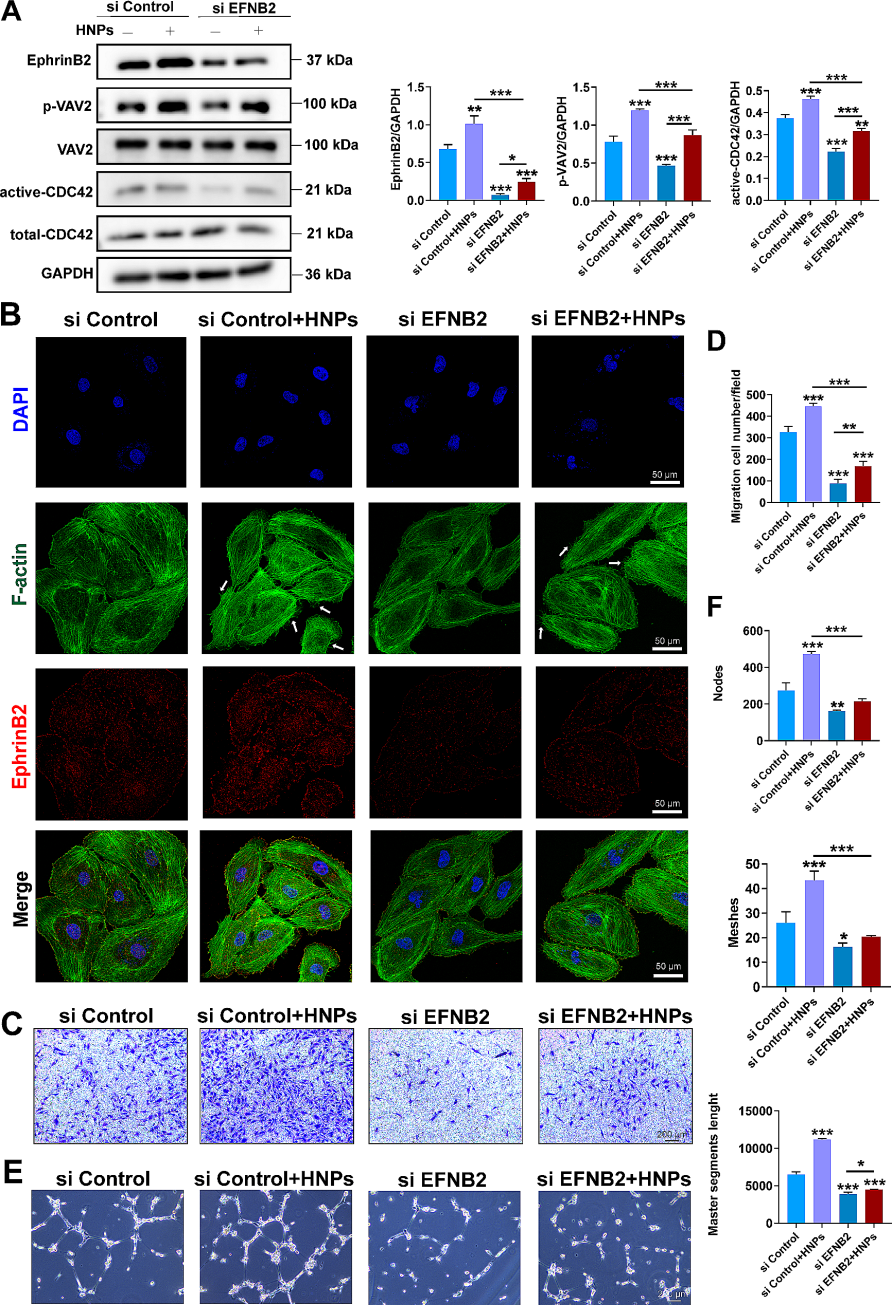
HNPs promote endothelial cell migration through EphrinB2. (**A**) Quantification of EphrinB2, p-VAV2, and active CDC42 protein levels. (**B**) Immunofluorescence images of cellular cytoskeletons captured using laser confocal microscopy. (**C-D**) Transwell experiment; (**E-F**) Tube formation experiment; quantitative analysis of node count, mesh count and master segment length using ImageJ (*P < 0.05; **P < 0.01; ***P < 0.001)

**Fig. 8:**
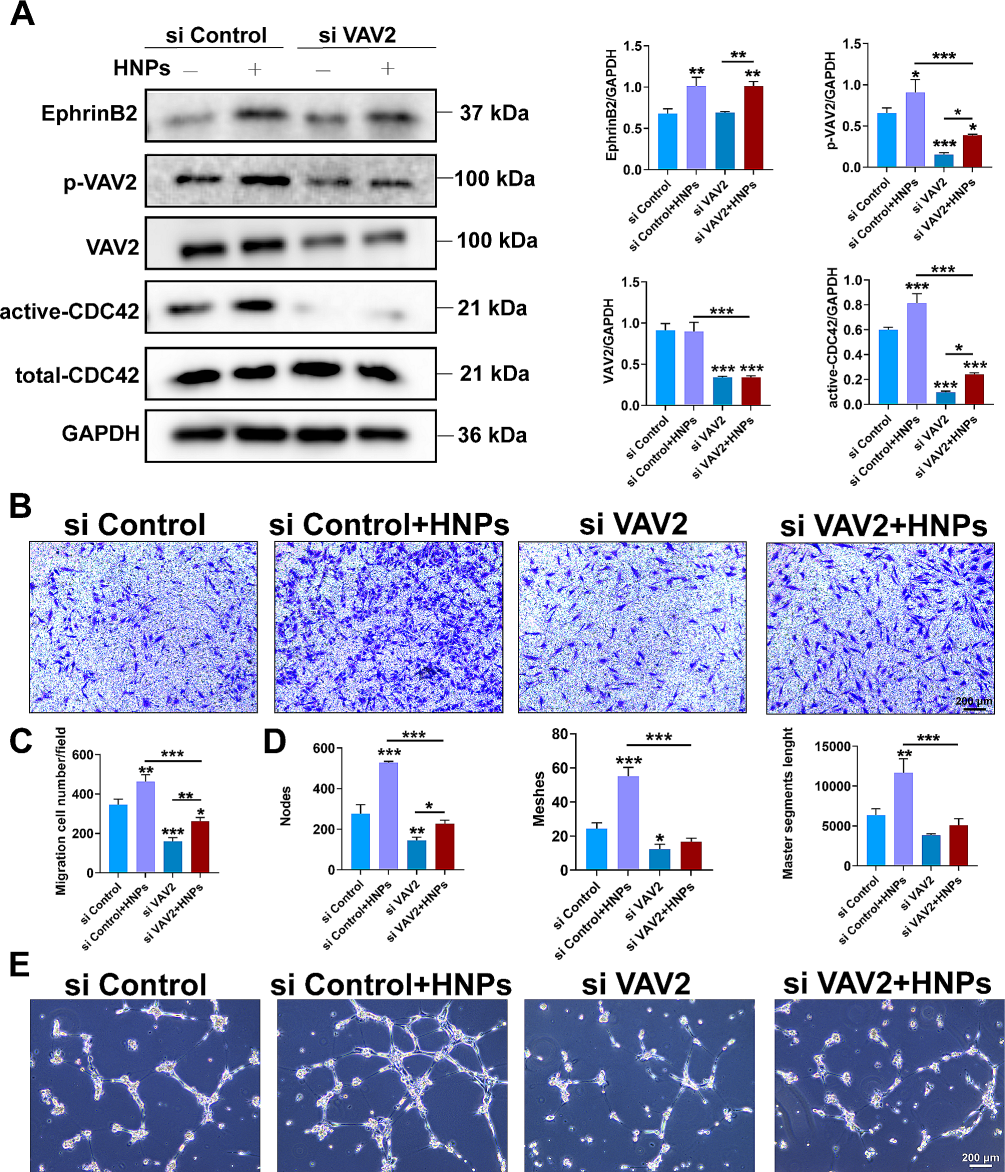
HNPs mediate endothelial cell migration through EphrinB2/VAV2/CDC42. (**A**) Protein expression levels of EphrinB2, p-VAV2, VAV2, and active-CDC42, with the quantitative results presented in histogram format. (**B**) Transwell images. (**C**) Quantitative analysis of the number of endothelial cells migrated to the lower chamber in each group, presented in histogram form. (**D-E**) Images of tube formation and quantitative analysis of node count, mesh count and master segment length using ImageJ (*P < 0.05; **P < 0.01; ***P < 0.001)

To investigate the regulatory effect of HNPs mediated by EphrinB2, we transfected EFNB2 siRNA into HUVECs. The WB results are shown in Fig. 7A. The EFNB2 gene was effectively knocked down in the siEFNB2 group compared to the control group (siControl). The levels of phosphorylated VAV2 and activated CDC42 were inhibited to varying degrees, while the total protein expression of VAV2 and CDC42 did not significantly change. This finding indicates that EphrinB2 can activate VAV2 and CDC42. Furthermore, HNPs rescued the expression of EphrinB2, further rescuing the downstream phosphorylation of VAV2 and activating CDC42. Immunofluorescence staining revealed that both the siControl group and the HNPs-treated group exhibited a more ordered cell skeleton arrangement and more robust filamentous pseudopodia protruding from the cells than the siEFNB2 group (Fig. 7B). In the Transwell experiment, the migration rate of cells in the siEFNB2 group was significantly lower than that in the siControl group, and when HNPs were added, the migration rate of HUVECs significantly increased (Fig. 7C, D). In the tube formation experiment, the siEFNB2 group showed a significant decrease in the node count, mesh count and master segment length. However, HNPs significantly increased these parameters (Fig. 7E, F). Moreover, after knockdown of VAV2, EphinB2 expression was not affected, but CDC42 expression decreased (Fig. 8A). Similarly, HUVECs had fewer filopodia and a more disorganized cytoskeleton (Fig. S6), showing a significant reduction in migration and tube formation (Fig. 8B-E). However, when HNPs were added, these adverse effects on angiogenesis were reversed. Taken together, these results indicate that HNPs can regulate endothelial cell migration through the EphrinB2/VAV2/CDC42 pathway, thereby promoting angiogenesis.

**Fig. 9:**
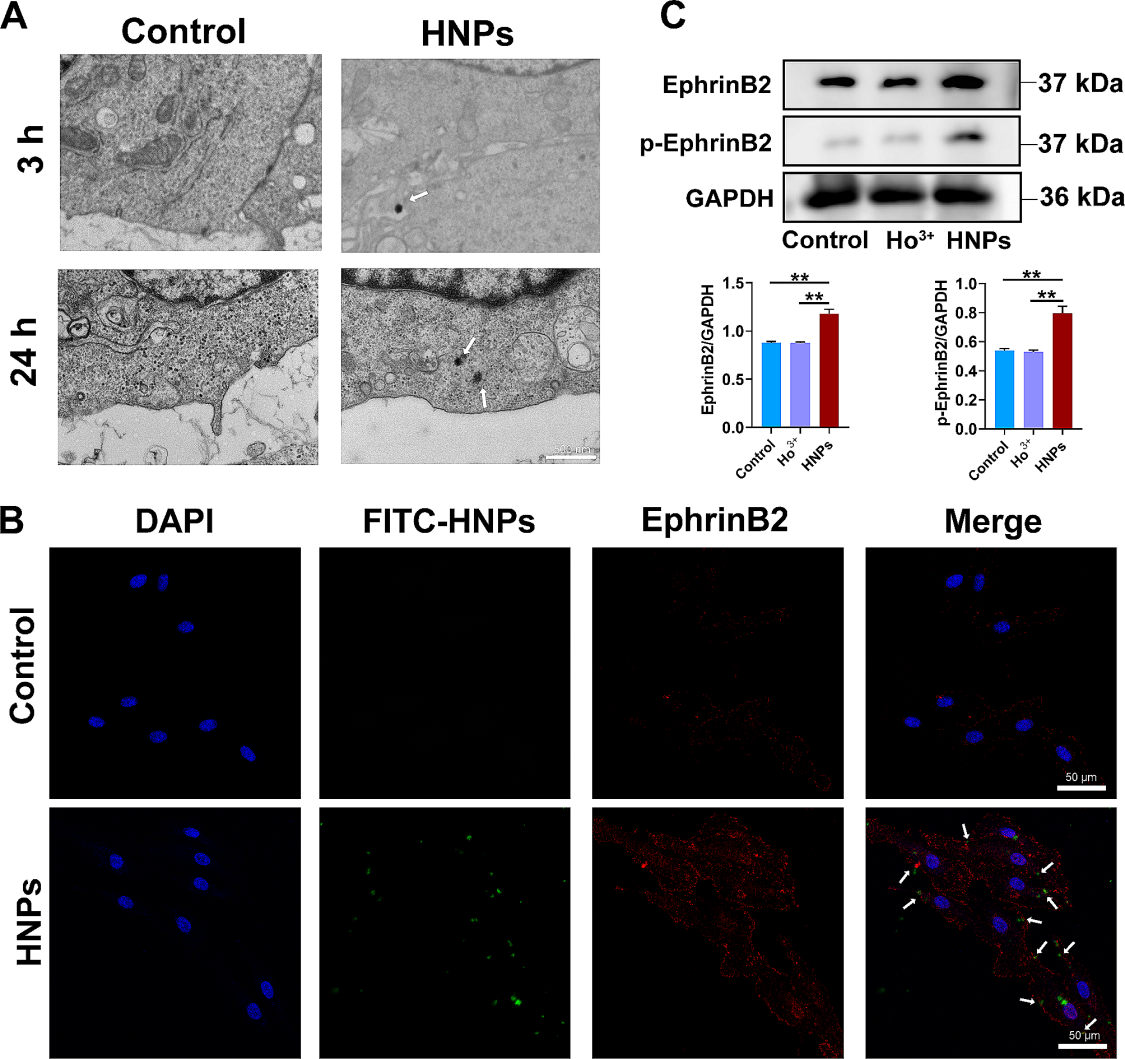
HNPs interact with EphrinB2. (**A**) Observation of the localization of HNPs through TEM. (**B**) Immunofluorescence analysis of the localization and expression of HNPs (green) and EphrinB2 (red) in cells. (**C**) Protein levels of EphrinB2 and phosphorylated EphrinB2 in the control group, the holmium ion solution group and the HNPs group determined by WB analysis (**P < 0.01)

However, how HNPs regulate the form and expression of EphrinB2 remains unclear. The exact mechanism by which metal nanoparticles induce intracellular biological events has not been fully elucidated. Notably, the interaction between nanoparticles and the cell membrane is a key factor influencing their biological effects. Under our experimental conditions, the contact with and internalization of HNPs by the cell membrane are the initial processes necessary for HNPs to promote vascular function. Through TEM, we observed the distribution of HNPs in HUVECs. In the early stage (3 h), HNPs were adsorbed to the cell membrane, and in the later stage (24 h), HNPs were internalized into the cytoplasm (Fig. 9A). Therefore, we hypothesize that HNPs interact with EphrinB2 on the cell membrane to activate and upregulate EphrinB2. To verify this hypothesis, First, we determined the localization of HNPs and EphrinB2 in cells through immunofluorescence experiments. FITC-labelled HNPs adhered to the cell membrane of HUVECs in the early stage and colocalized with EphrinB2 on the cell membrane (Fig. 9B). In addition, we cultured HUVECs with a holmium ion solution, and WB assay revealed no notable variances in the expression levels of EphrinB2 or phosphorylated EphrinB2 (Fig. 9C). Combining this information, we concluded that phosphorylated EphrinB2 was activated by the interaction between HNPs and EphrinB2.

Thus far, based on transcriptomics and experimental results in vitro and in vivo, we speculate that HNPs can interact with the cell membrane (the specific pathway involved is unclear), thereby inducing the cascade activation of the EphrinB2/VAV2/CDC42 signalling pathway, regulating endothelial cell migration, and promoting angiogenesis.

## Discussion

Promoting angiogenesis through biomaterial implantation is a key strategy in addressing the challenge of reconstructing large segmental bone defects. Nanomaterials are easier to use in tissue engineering scaffolds due to their size effects and surface physicochemical properties [37]. The HNPs used in this study have an average size of 50 nm, and the surface is rich in chemical groups, which are a type of LM NPs with excellent properties. Our study showed that HNPs can promote angiogenesis and bone tissue repair and the activation of the EphrinB2/VAV2/CDC42 cascade promotes angiogenesis. These phenomena and mechanisms are currently unique or have not been described for LM NPs.

Recently, LM NPs have been shown to be promising angiogenic materials, as they mainly promote angiogenesis by regulating ROS. Lanthanum hydroxide nanoparticles can reduce oxidative stress by eliminating excess ROS from cells and activating the VEGF signalling pathway [38]. However, neodymium oxide nanoparticles promote angiogenesis by producing small amounts of ROS/reactive nitrogen species (RNS) [32]. Defining and accurately regulating appropriate ROS levels in cells is difficult, and the ultimate angiogenic effect is affected by many factors, such as reaction time, temperature, and pH [39]. These factors limit the application of the lanthanide nanoparticles mentioned above. In contrast to the vascular regulatory mechanism of lanthanide nanomaterials mentioned above, our study revealed that HNPs promote vascular regeneration by directly regulating the migratory process rather than regulating the intracellular ROS content. On one hand, this finding may be because the surface of the HNPs used in this study does not have multivalent atoms or sufficient oxygen vacancies or because the particle size is still large relative to the catalytically highly active ultra-small micro nanoparticles. On the other hand, protein adsorption on the particle surface may alter the physicochemical properties. Our study also suggests that LM MPs may have diverse mechanisms of action that are not limited to ROS regulation. Future studies should explore whether other LM MPs also act through EphrinB2/VAV2/CDC42 or similar signalling pathways and evaluate their role in angiogenesis.

According to the sequencing results, we found that the biological functions related to endothelial cell migration were enriched. Through TEM and immunofluorescence experiments, we observed that HNPs were localized early on the membrane and colocalized with EphrinB2, a migration-related membrane protein. The expression of EphrinB2 and phosphorylated EphrinB2 did not increase when HNPs were replaced with holmium nitrate ion solution, indicating that HNPs can interact with and activate EphrinB2. This finding may be related to the “particle specificity” of metal nanoparticles or the “protein corona” formed by surface adsorption [40, 41]. The HNPs in this study are rich in alkaline hydroxyl groups, which can be easily esterified with carboxyl groups on the protein surface and promote interactions with proteins. However, systematic research on the effects of nanoparticles on membrane proteins in cell membranes is lacking. By interacting with membrane proteins, nanoparticles can more accurately locate and act on target cells, thereby regulating their biological functions. Therefore, EphrinB2 serve as an important target protein regulating angiogenesis.

Previous studies have shown that Tyr304 phosphorylation of the C-terminal domain of EphrinB2 can bind with high affinity to the Src homeodomain 2 (SH2) domain of Grb4 to play a role in signal transduction [36]. Unlike that of any other Ras superfamily GEFs, the SH2 domain is a unique structural feature of a VAVs, so we speculate that VAV2 may bind with EphrinB2 [35]. In the CO-IP assay, VAV2 could be detected by using EphrinB2 as the target antigen and vice versa. Molecular simulations further confirmed the interaction of EphrinB2 with VAV2, where hydrogen bonding promotes a strong non-covalent interaction between EphrinB2 and VAV2. By co-immunoprecipitation and molecular simulation, we confirmed their bind for the first time. VAV2 activates CDC42, an important small GTP enzyme in the Rho family, by catalysing GDP release and GTP binding [42]. CDC42 regulates cytoskeletal recombination and extends filamentous pseudopods, promoting migration [43]. Unlike stress fibres, filamentous pseudopods are slender projections composed of actin filaments that more effectively regulate the efficiency and direction of cell migration [44]. After transfection with siEFNB2 and siVAV2, filopodia were inhibited. Therefore, EphrinB2/VAV2/CDC42 are important targets for regulating angiogenesis, and HNPs can also be used in tissue engineering as membrane-affecting nanoparticles.

HNPs are a special kind of LM NPs. Holmium has also been reported to have superparamagnetic and upconversion luminescence properties, and composite materials designed accordingly have the dual functions of bone repair and multimodal imaging, as well as the potential for use as “biomarkers“ [45, 46]. In the future, along with the superior angiogenic effect of HNPs we reported, tissue engineering nanomaterials with multiple functions can be designed. To promote the biological application of HNPs, researchers should further study the dynamic distribution and metabolism of HNPs in vivo. Surface functional retouching and generation of slow-release systems can also expand these applications.

## Conclusion

In this study, for the first time, we demonstrated that HNPs exhibit good angiogenic properties, creating a favourable vascular microenvironment to promote bone repair. Additionally, we found that HNPs can interact with the cell membrane early on, activating the membrane protein EphrinB2, which cascades to activate VAV2/CDC42. The EphrinB2/VAV2/CDC42 signalling pathway regulates endothelial cell migration and promotes angiogenesis. Overall, our research demonstrated the superior angiogenic properties of HNPs, providing new insights into their biological functions. HNPs can be considered a new candidate biomaterial for promoting bone tissue engineering repair.

### Electronic supplementary material

Below is the link to the electronic supplementary material.

Supplementary Material 1

## Data Availability

The data that support the findings of this study are available from the corresponding author upon reasonable request.
