# Supplementary material for: Proangiogenic effect and underlying mechanism of holmium oxide nanoparticles: a new biomaterial for tissue engineering
Source: J Nanobiotechnology. 2024 Jun 21;22:357. doi: 10.1186/s12951-024-02642-x (PMC11191282; doi:10.1186/s12951-024-02642-x)
Supplement: Supplementary file 1 — Supplementary Material 1 [file 12951_2024_2642_MOESM1_ESM.docx]

**Supporting Information**

**Proangiogenic effect and underlying mechanism of holmium oxide nanoparticles: A new biomaterial for tissue engineering**

Yuxiao Luo ^1^, Yifan Zheng ^1^, Ziwei Chen ^2^, Minhua Mo ^2^, Jiling Xie ^2^, Xiaohe Zhou ^2^, Yupeng Wu^2^, Qiyuan Yang^2^, Manjia Zheng^2^, Xiaowen Hu^2^, Liangjiao Chen ^2, *^, Zedong Lan ^1, *^

1. Shenzhen Stomatological Hospital, Southern Medical University, Shenzhen, Guangdong 518001, People’s Republic of China.

2. Department of Orthodontics, School and Hospital of Stomatology, Guangdong Engineering Research Center of Oral Restoration and Reconstruction & Guangzhou Key Laboratory of Basic and Applied Research of Oral Regenerative Medicine, Guangzhou Medical University, Guangzhou, China.

* Corresponding author:

Zedong Lan, [lanzedong@smu.edu.cn](mailto:lanzedong@smu.edu.cn)

Liangjiao Chen, [2010686017@gzhmu.edu.cn](mailto:2010686017@gzhmu.edu.cn)

**Supplementary Figures**

**
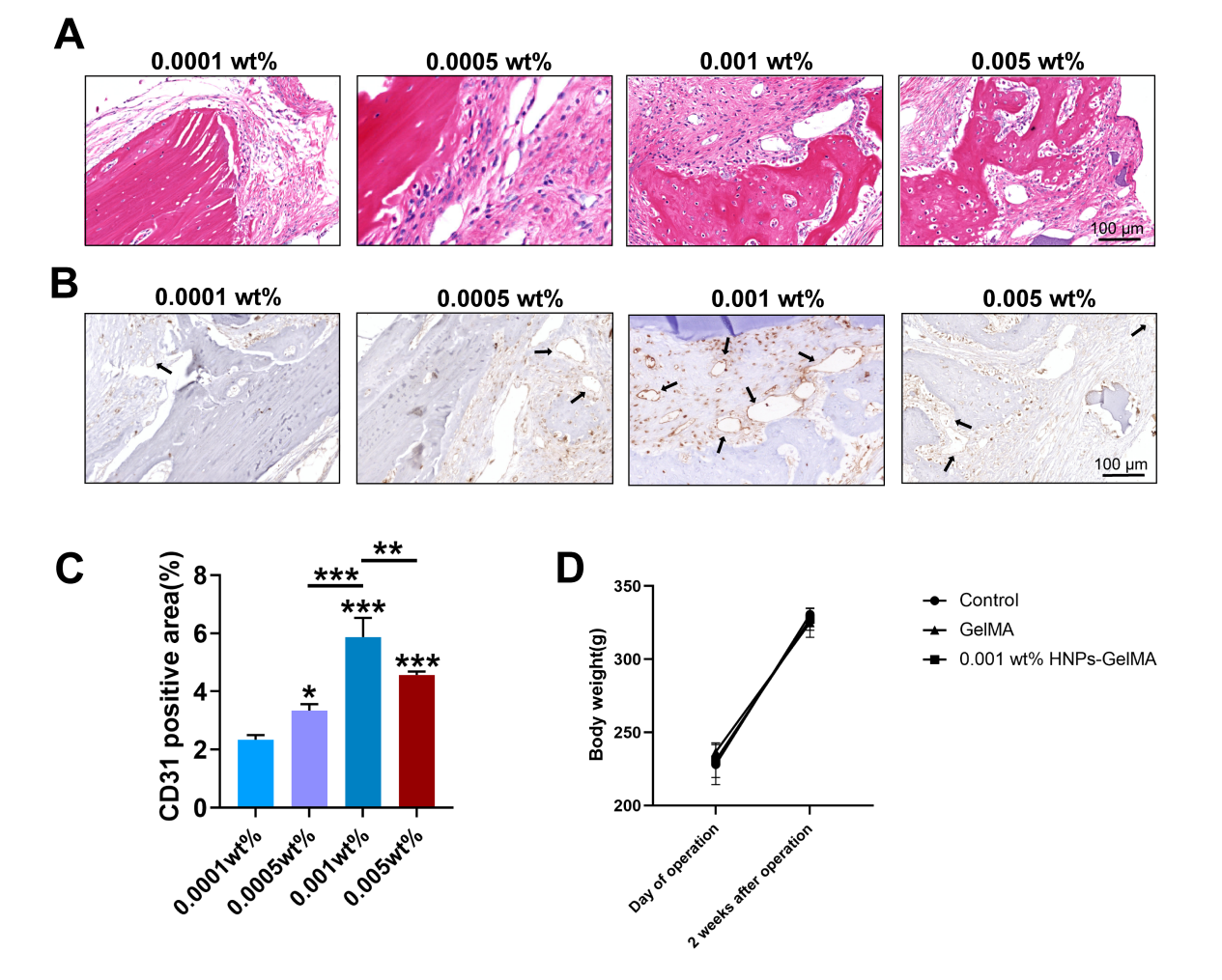
**

**Fig. S1.** Area of bone defect model after 2 weeks of treatment. **A.** HE staining. **B.** CD31 immunohistochemical staining. **C.** Quantitative analysis of CD31-positive areas, showing a significant increase in neovascularization in the 0.001wt% HNPs group compared to other groups. **D.** Body weight change in operative period (**P* < 0.05; ***P* < 0.01; ****P* < 0.001).

**
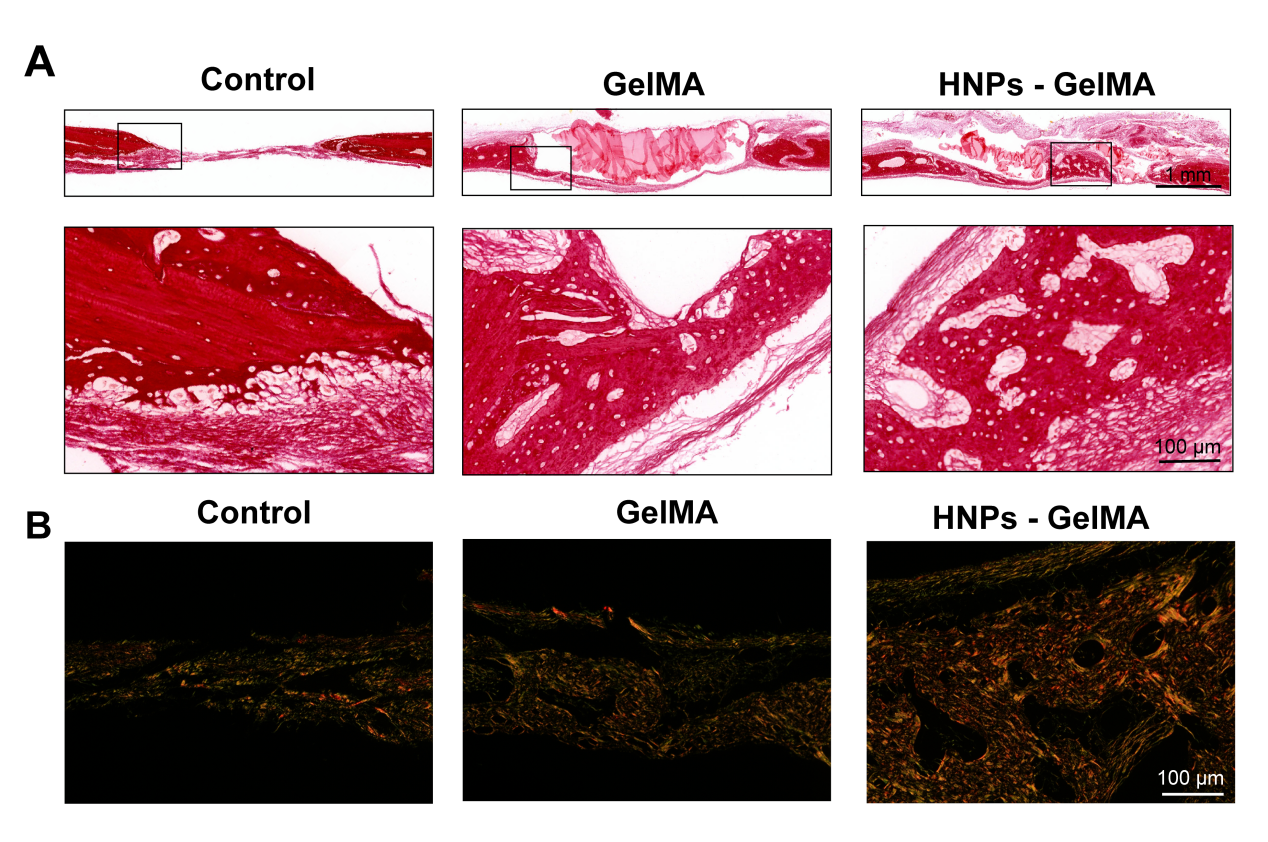
**

**Fig. S2.** The rat cranial bone defect stained with Sirius Red. **A.** Sirius red staining images and **B.** Polarized light images both showed there were more type I collagens and their arrangement was more regular in HNPs-GelMA group.

**
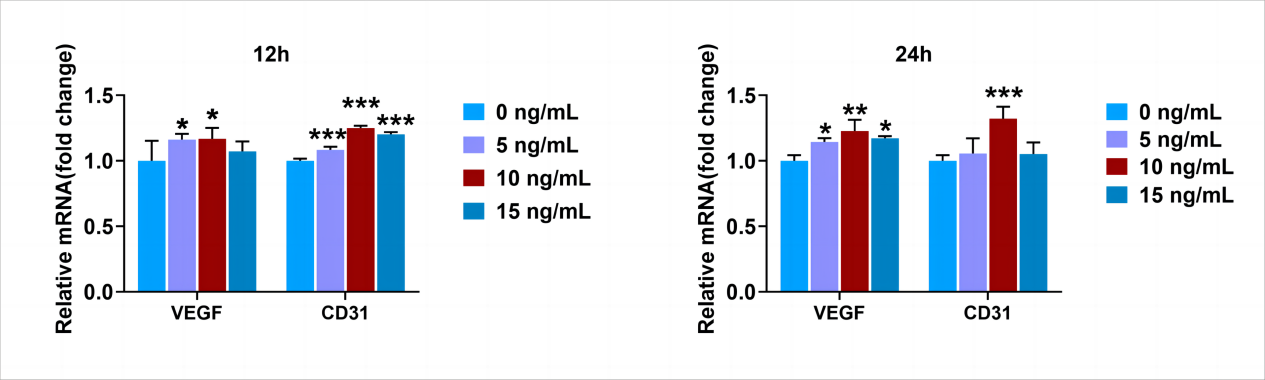
**

**­­Fig. S3.** Quantitative analysis of gene expression related to angiogenesis. VEGF and CD31 mRNA after treatment with 0, 5, 10, or 15 ng/mL HNPs for 12 and 24 hours. Compared to other groups, the 10 ng/mL group showed the highest expression levels of VEGF and CD31 mRNA (**P* < 0.05; ***P* < 0.01; ****P* < 0.001).

**
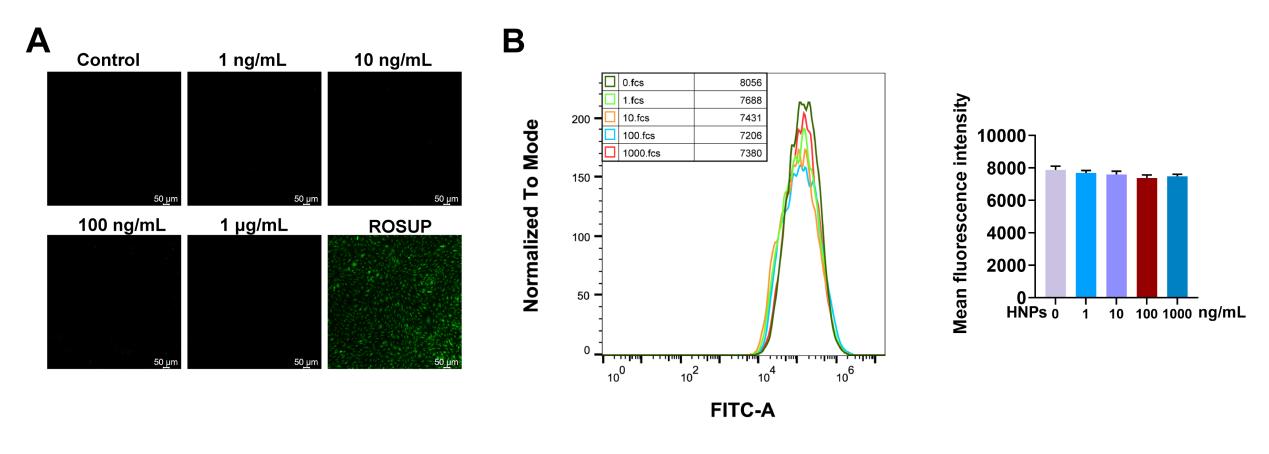
**

**Fig. S4.** ROS detection in HUVECs treated with HNPs **A.** Images of intracellular ROS in HUVECs captured using laser confocal microscopy. **B.** Detection of ROS in HUVECs using flow cytometry.

**
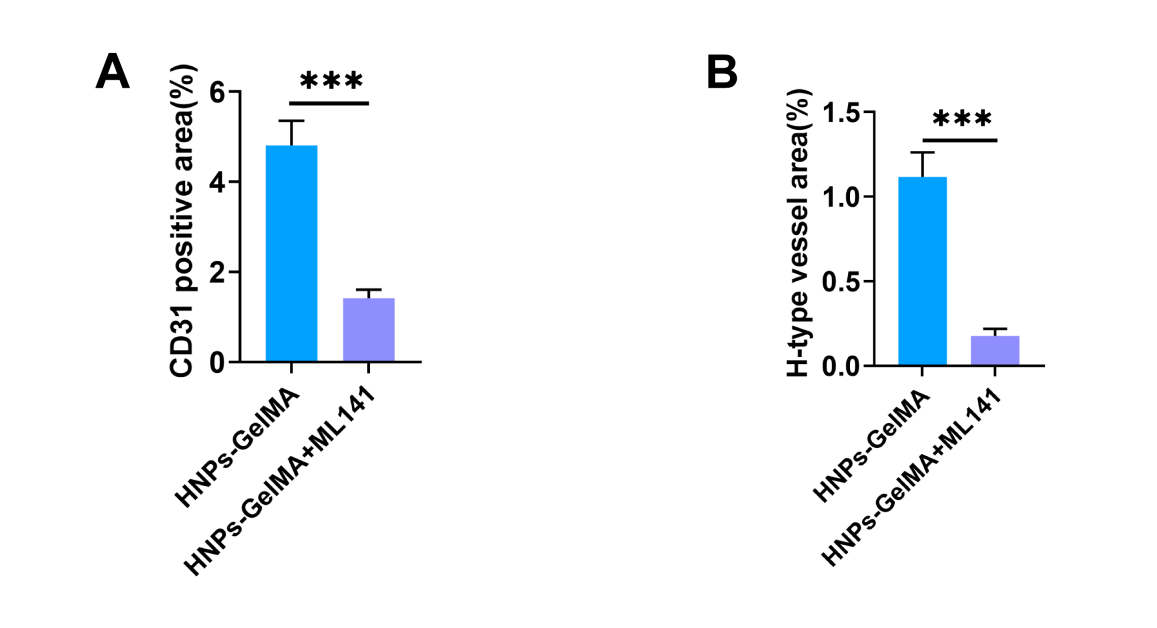
**

**­­Fig. S5.** The role of CDC42 in HNPs-promoted vascular regeneration. **A.** Quantitative analysis of positive areas after CD31 immunohistochemical staining. **B.** Quantitative analysis of H-type vascular volume (****P* < 0.001).

**
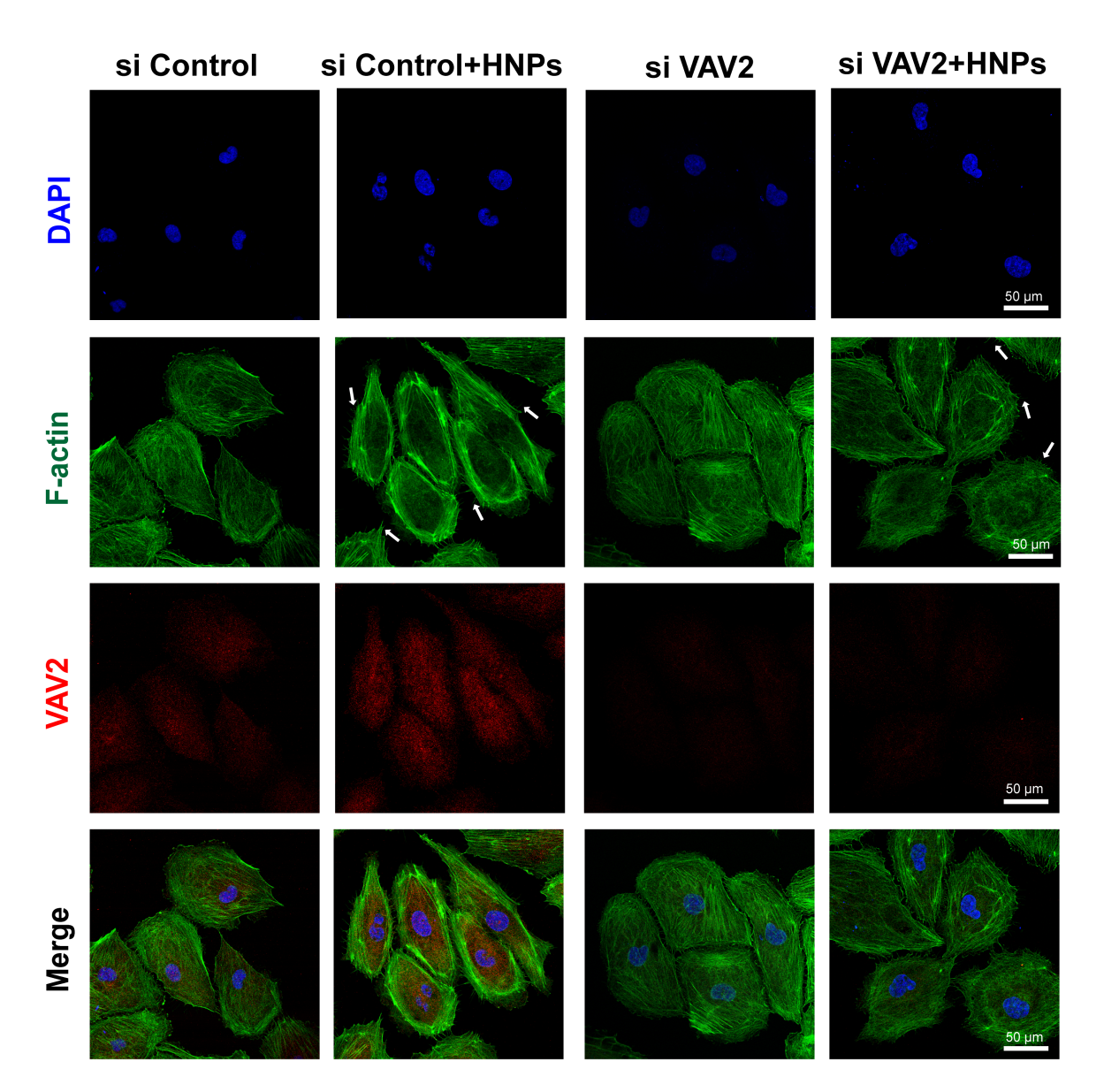
**

**­­Fig. S6.** HNPs promote endothelial cell migration through Ephrin B2/VAV2/CDC42. Immunofluorescence images of cellular cytoskeletons captured using laser confocal microscopy (the arrows represent filopodia).

**Supplementary Tables**

**Table S1.** Primers used for RT- qPCR (F: forward primer R: reverse primer)

| **Gene** | **Primer** | **Primer nucleotide sequence (5’-3’)** |
| --- | --- | --- |
| **GAPDH** | F | AATTCCATGGCACCGTCAAGG |
|  | R | GAGGGATCTCGCTCCTGG |
| **CD31** | F | GACGATGTCAGAAACCATGCAA |
|  | R | GTCCTTCTTTCCTAGATCTTTGTGA |
| **VEGF** | F | ACAAATGTGAATGCAGACCAAA |
|  | R | ACCAACGTACACGCTCCAG |
| **EFNB2** | F | AAGGACTGGTACTATACCCACAG |
|  | R | TGTCTGCTTGGTCTTTATCAACC |

**Table S2.** Primers used for transfection (F: forward primer R: reverse primer)

| **Gene** | **Primer** | **Primer nucleotide sequence (5’-3’)** |
| --- | --- | --- |
| **si EFNB2-1** | F | GGUACUAUACCCACAGAUAGGTT |
|  | R | CCUAUCUGUGGGUAUAGUACCTT |
| **si EFNB2-2** | F | GCAGACAGAUGCACUAUUAAGTT |
|  | R | CUUAAUAGUGCAUCUGUCUGCTT |
| **si EFNB2-3** | F | AGAAGAACAAAGAUUAUUACATT |
|  | R | UGUAAUAAUCUUUGUUCUUCUTT |
| **si** **VAV2-1** | F | GGUACUUGUUCCUGUUUGACATT |
|  | R | UGUCAAACAGGAACAAGUACCTT |
| **si VAV2-2** | F | AGGUCUUCCUCGAUUUCAAGGTT |
|  | R | CCUUGAAAUCGAGGAAGACCUTT |
| **si VAV2-3** | F | CAGAGGAGACCACAGAGAAUGTT |
|  | R | CAUUCUCUGUGGUCUCCUCUGTT |
| **Negative control** | F | UUCUCCGAACGUGUCACGUTT |
|  | R | ACGUGACACGUUCGGAGAATT |

**Table S3**. Free energy of binding of Ephrin B2 to VAV2

| **RESIDUE_ID** | **VDW** | **ELE** | **GB** | **SA** | **TOTAL** |
| --- | --- | --- | --- | --- | --- |
| lig-rec | -71.37 | -170.49 | 228.32 | -10.44 | -23.97 |
| R-A-ASN-107 | -3.21 | -8.29 | 6.29 | -0.5 | -5.71 |
| R-A-PRO-17 | -1.67 | -3.46 | 2.88 | -0.41 | -2.66 |
| R-A-GLY-108 | -0.62 | -3.41 | 1.51 | -0.09 | -2.61 |
| R-A-GLU-111 | -1.38 | -34.67 | 34.11 | -0.53 | -2.48 |
| R-A-ARG-124 | -2.13 | -47.5 | 48.28 | -0.41 | -1.75 |
| R-A-LYS-41 | -2.25 | -29.32 | 30.29 | -0.32 | -1.6 |
| R-A-VAL-43 | -2.23 | 27.91 | -26.64 | -0.55 | -1.49 |
| R-A-SER-40 | 1.12 | -1.88 | 1.9 | -0.28 | -1.38 |
| R-A-ASN-115 | -0.64 | -5.48 | 5.1 | -0.21 | -1.24 |
| R-A-SER-109 | -2.32 | 4.35 | -2.76 | -0.33 | -1.05 |
| L-B-ARG-45 | -0.66 | -40.19 | 36.24 | -0.3 | -4.92 |
| L-B-PRO-46 | -3.79 | -2.03 | 2.04 | -0.7 | -4.47 |
| L-B-ASN-22 | -3.04 | -8.3 | 8.45 | -0.93 | -3.82 |
| L-B-SER-117 | -2.92 | -1.33 | 3.42 | -0.47 | -1.3 |
| L-B-PHE-19 | -1.26 | 0.36 | 0.06 | -0.26 | -1.1 |
| L-B-GLY-118 | -1.46 | -0.17 | 0.99 | -0.35 | -0.99 |

Note: Units are in kcal/mol, where residues with a binding energy of <-1 kcal/mol are defined as binding hotspots. Lig is ligand, rec is receptor. ∆Eelectrostatic (electrostatic), and ∆Evdw (van der Waals) energies. ∆Gsolv is the sum of electrostatic solvation energy (polar contribution), ∆GPB/GB, and the nonelectrostatic solvation component (nonpolar contribution), ∆GSA.
